# Soluble Epoxide Hydrolase Inhibition by *t*-TUCB Promotes Brown Adipogenesis and Reduces Serum Triglycerides in Diet-Induced Obesity

**DOI:** 10.3390/ijms21197039

**Published:** 2020-09-24

**Authors:** Haley Overby, Yang Yang, Xinyun Xu, Katherine Graham, Kelsey Hildreth, Sue Choi, Debin Wan, Christophe Morisseau, Darryl C. Zeldin, Bruce D. Hammock, Shu Wang, Ahmed Bettaieb, Ling Zhao

**Affiliations:** 1 Department of Nutrition, University of Tennessee, Knoxville, TN 37996, USA; haleyoverbyphd@gmail.com (H.O.); yyang100@vols.utk.edu (Y.Y.); xxu28@vols.utk.edu (X.X.); kgraha19@vols.utk.edu (K.G.); khildre2@vols.utk.edu (K.H.); schoi20@vols.utk.edu (S.C.); 2 Department of Entomology and Nematology, and Comprehensive Cancer Center, University of California, Davis, CA 95616, USA; dbwan@ucdavis.edu (D.W.); chmorisseau@ucdavis.edu (C.M.); bdhammock@ucdavis.edu (B.D.H.); 3 Division of Intramural Research, National Institute of Environmental Health Sciences, Research Triangle Park, NC 27709, USA; zeldin@niehs.nih.gov; 4 Department of Nutritional Sciences, Texas Tech University, Lubbock, TX 79409, USA; shu.wang@ttu.edu; 5 Graduate School of Genome Science and Technology, University of Tennessee, Knoxville, TN 37996, USA

**Keywords:** soluble epoxide hydrolase, soluble epoxide hydrolase inhibitor, *t*-TUCB, brown adipose tissue, brown adipogenesis

## Abstract

Brown adipose tissue (BAT) is an important target for obesity treatment and prevention. Soluble epoxide hydrolase (sEH) converts bioactive epoxy fatty acids (EpFAs) into less active diols. sEH inhibitors (sEHI) are beneficial in many chronic diseases by stabilizing EpFAs. However, roles of sEH and sEHI in brown adipogenesis and BAT activity in treating diet-induced obesity (DIO) have not been reported. sEH expression was studied in in vitro models of brown adipogenesis and the fat tissues of DIO mice. The effects of the sEHI, *trans*-4-{4-[3-(4-trifluoromethoxy-phenyl)-ureido]-cyclohexyloxy-benzoic acid (*t*-TUCB), were studied in vitro and in the obese mice via mini osmotic pump delivery. sEH expression was increased in brown adipogenesis and the BAT of the DIO mice. *t*-TUCB promoted brown adipogenesis in vitro. Although *t*-TCUB did not change body weight, fat pad weight, or glucose and insulin tolerance in the obese mice, it decreased serum triglycerides and increased protein expression of genes important for lipid metabolism in the BAT. Our results suggest that sEH may play a critical role in brown adipogenesis, and sEHI may be beneficial in improving BAT protein expression involved in lipid metabolism. Further studies using the sEHI combined with EpFA generating diets for obesity treatment and prevention are warranted.

## 1. Introduction

Brown adipose tissue (BAT) has recently emerged as a novel target for obesity treatment/prevention [1,2,3]. In contrast to white adipose tissue (WAT), BAT is responsible for non-shivering thermogenesis through uncoupling ATP synthesis from respiration via uncoupling protein 1 (UCP1), leading to dissipation of energy as heat [1]. It was long believed that BAT was transient within the first months of life and then drastically reduced in adulthood. Using positron emission tomography/computed tomography (PET/CT) imaging, it was recently discovered that adult humans do have a significant amount of functional BAT [4,5,6,7]. These depots are either classical brown or beige fat [8,9,10]. Beige fat or brown-like fat is a type of fat that is formed in the WAT depot by a “browning” process in response to various stimuli [11,12]. Current strategies to enhance functional BAT mass/activity include cold exposure and β-adrenergic stimulation; however, these strategies have issues of compliance and side effects. Novel effective strategies are needed for obesity treatment and prevention.

Soluble epoxide hydrolase (sEH, encoded by *Ephx2* gene) is a predominantly cytosolic enzyme that converts epoxy fatty acids (EpFAs) into less active diols by adding a water molecule [13]. EpFAs are produced by cytochrome P-450 enzymes (CYP450) from n-6 polyunsaturated fatty acids (PUFA), such as arachidonic acid (AA) and n-3 PUFA, such as docosahexaenoic acid (DHA) and eicosapentaenoic acid (EPA). Many EpFAs are autocrine/paracrine lipid signaling molecules that play essential roles in pain, inflammation, vascular dilation, and cell growth/differentiation [13]; therefore, sEH, expressed in various tissues, including white adipocytes and WAT [14], has become a pharmacological target. Potent small molecule sEH inhibitors have been developed to stabilize endogenous EpFAs and enhance their beneficial effects. 

sEH inhibition and/or sEH deficiency have been shown to decrease ER stress [15] and inflammation [16] in the WAT and liver in diet-induced obesity (DIO) and associated liver steatosis [16], cardiac remodeling [17], and endothelial dysfunction [18]. Interestingly, one study showed that an sEH inhibitor induced weight loss in high fat-high fructose-fed obese mice, which was associated with increased heat production and UCP1 protein expression in the interscapular BAT (iBAT) [19]. In another study, a different sEH inhibitor significantly increased the iBAT mass in the *fat-1* mice [20], which had transgenic expression of a n-3 desaturase, leading to enriched endogenous n-3 PUFA levels and higher n-3 PUFA-derived EpFAs [20]. However, the regulation of sEH expression in the iBAT in DIO and the in vitro models of brown adipogenesis have not been directly studied. Moreover, whether an sEH inhibitor acts in a cell-autonomous manner to promote brown adipogenesis, enhances iBAT activity and improves metabolic dysfunction in DIO have not been investigated. 

In the current study, we investigated sEH expression in in vitro models of brown adipogenesis of murine and human origins and in the WAT and iBAT of diet-induced obese C57BL/6J mice. Moreover, the effects of sEH inhibition by *trans*-4-{4-[3-(4-trifluoromethoxy-phenyl)-ureido]-cyclohexyloxy-benzoic acid (*t*-TUCB) were studied in the murine brown adipogenesis model and obese C57BL/6J mice via osmotic pump delivery. 

## 2. Results

### 2.1. sEH Expression Is Increased in In Vitro Models of Brown Adipogenesis and the Fat Tissues in Diet-Induced Obesity

**Figure 1:**
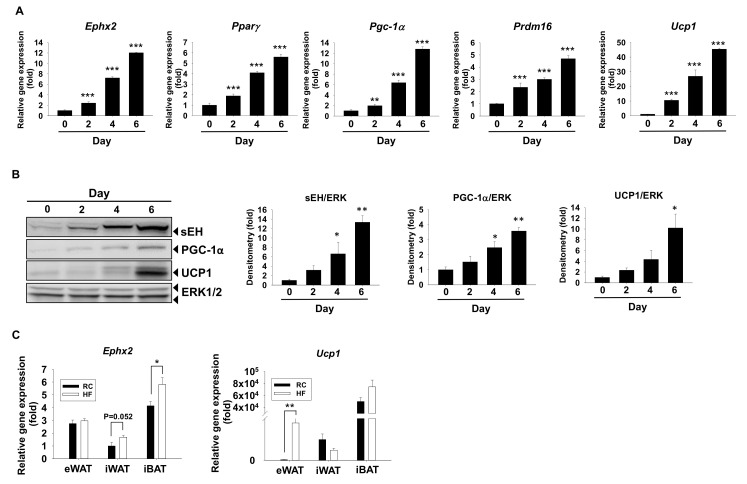
sEH mRNA and protein expression are increased during murine brown adipogenesis in vitro. (**A**,**B**) Murine brown preadipocytes were induced to differentiate for 6 days. Total RNA samples were collected at day 0 (0), day 2 (2), day 4 (4), and day 6 (6). (**A**) Relative mRNA levels of brown marker gene *Pparγ*, *Pgc-1α*, *Ucp1*, *Prdm16*, and *Ephx2*. mRNA expression was quantified relatively to the loading control *36b4*. (**B**) Protein expression of sEH, PGC-1α, UCP1, and the loading control extracellular signal-regulated kinases (ERK1/2). Bar graphs show normalized densitometry for sEH/ERK1/2, PGC-1α/ERK1/2, and UCP1/ERK1/2. Data = Mean + SEM (n = 3). (**C**) Changes in *Ephx2* mRNA levels in white and brown fat tissue in diet-induced obesity. Six-weeks old C57BL/6J mice were fed with either a high-fat diet (60% kcal from fat) (HF) or a regular chow diet (RC) for 20 weeks, then sacrificed at 26 weeks of age. eWAT, iWAT, and iBAT were collected, total RNA was isolated, and mRNA levels of *Ephx2* and *Ucp1* were analyzed by semi-quantitative RT-PCR. Relative mRNA expression levels of *Ephx2* or *Ucp1* of iWAT from RC group were set to be fold 1. Data = Mean + SEM (n = 6). *, **, ***, *p* < 0.05, *p* < 0.01 or *p* < 0.001 compared to the day 0 sample (**A**,**B**) or the control group (**C**), respectively.

First, sEH mRNA and protein expression were examined during murine brown adipogenesis in vitro. mRNA expression of sEH encoding *Ephx2* was time-dependently increased, along with brown marker peroxisome proliferator-activated receptor gamma (*Pparγ*), peroxisome proliferator-activated receptor gamma coactivator 1-alpha (*Pgc-1α*), PR domain containing 16 (*Prdm16*), and *Ucp1* during differentiation (Figure 1A). Consistently, sEH protein expression was also time-dependently increased, along with PGC-1α and UCP1 protein expression during the process (Figure 1B). 

To gain insights into the role of sEH in the development of obesity in mice, *Ephx2* mRNA expression was also examined in various WAT pads and iBAT pad of the DIO mice (Figure 1C). Compared to the controls (regular chow or RC), *Ephx2* mRNA level was significantly increased in the iBAT of high fat-fed obese C57BL/6J mice (HF) (*p* < 0.05), but was not changed in the epididymal WAT (eWAT) (*p* < 0.05) (Figure 1C). sEH mRNA level was also increased in the inguinal WAT (iWAT) of the obese mice; however, the differences did not reach statistical significance (*p* = 0.0524) (Figure 1C). In contrast, there were no significant differences in *Ucp1* mRNA levels in the iBAT and iWAT between the RC and HF groups, although there were increases of *Ucp1* mRNA levels in the eWAT of the HF group (*p* < 0.01) (Figure 1C). 

**Figure 2:**
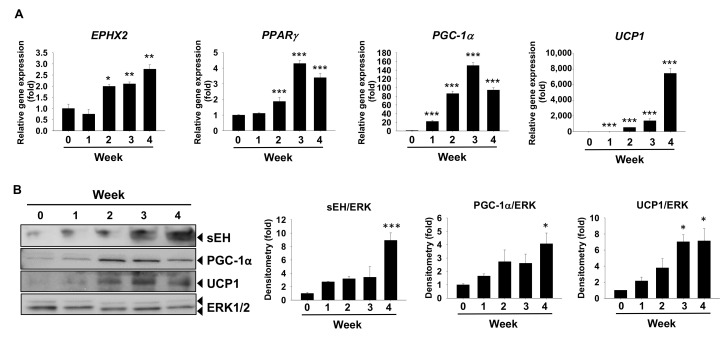
sEH mRNA and protein expression are increased during human brown adipogenesis in vitro. (**A**,**B**) Human brown preadipocytes were induced to differentiate for four weeks. Total RNA samples were collected at the start and the end of week 1, 2, 3, and 4 of the differentiation. (**A**) Relative mRNA levels of brown marker gene *PPARγ*, *PGC-1α*, *UCP1*, and *EPHX2*. mRNA expression was quantified relatively to the loading control 36B4 (**B**) Protein expression of sEH, PGC-1A, UCP1, and the loading control ERK1/2. Bar graphs show normalized densitometry for sEH/ERK1/2, PGC-1α/ERK1/2, and UCP1/ERK1/2. Data = Mean + SEM (n = 3). *, **, ***, *p* < 0.05, *p* < 0.01, and *p* < 0.001 compared to the week 0 sample, respectively.

Next, *EPHX2* mRNA and protein expression were examined during human brown adipogenesis in vitro. Similar to our observations in murine cells, *EPHX2* mRNA levels were also time-dependently increased during the process, along with mRNA levels of brown marker gene *PPARγ*, *PGC-1α*, and *UCP1* (Figure 2A). Protein expression of sEH, PGC-1α, and UCP1 were consistently increased along with their mRNA upregulation (Figure 2B).

We performed oxylipin analysis to gain insights on the sEH activities during brown adipocyte differentiation. The ratios of fatty acid diols over their parental epoxy fatty acids were calculated as markers of sEH activities, as reported [18,21]. Since we could not detect oxylipins in the cell extracts, we report the results from the media (Table S1) (The absolute data is also provided in the Supplemental Excel Sheets). The ratios of 9, 10-DiHOME/9, 10-EpOME (*p* < 0.05 for 12 h samples) and 12, 13-DiHOME/12, 13-EpOME (*p* < 0.05 for 12 h samples) as linoleic acid (LA) metabolites were significantly increased in the media from the differentiated murine brown adipocytes; Similarly, the ratios of 12, 13-DiHODE/12, 13-EpODE (*p* < 0.05 for 24 h samples) and 15, 16-DiHODE/15, 16-EpODE (*p* < 0.05 for 24 h samples) as alpha-linolenic acid (ALA) metabolites, and the ratios of 7, 8-DiHDPE/7, 8-EpDPE as docosahexaenoic acid (DHA) metabolites *(p* < 0.05 for 12 h samples) were also significantly increased in the differentiated brown adipocytes than the preadipocytes (Table S1). These results indicate higher sEH activities in the differentiated brown adipocytes than in the preadipocytes, consistent with higher sEH protein expression found in the differentiated murine brown adipocytes. 

In comparison, only the ratios of 7, 8-DiHDPE/7, 8-EpDPE (*p* < 0.05 for all 12, 24, and 48 h samples) and 19, 20-DiHDPE/19, 20-EpDPE (*p* < 0.01 for 12 h and 24 h samples) derived from DHA were significantly higher in differentiated human brown adipocytes than those in the preadipocytes (Table S1). 

### 2.2. sEH Inhibition by t-TUCB Promotes Murine Brown Adipogenesis, Upregulates Mitochondrial Respiratory Chain and Lipid Metabolic Genes, and Increases Mitochondrial Uncoupling

**Figure 3:**
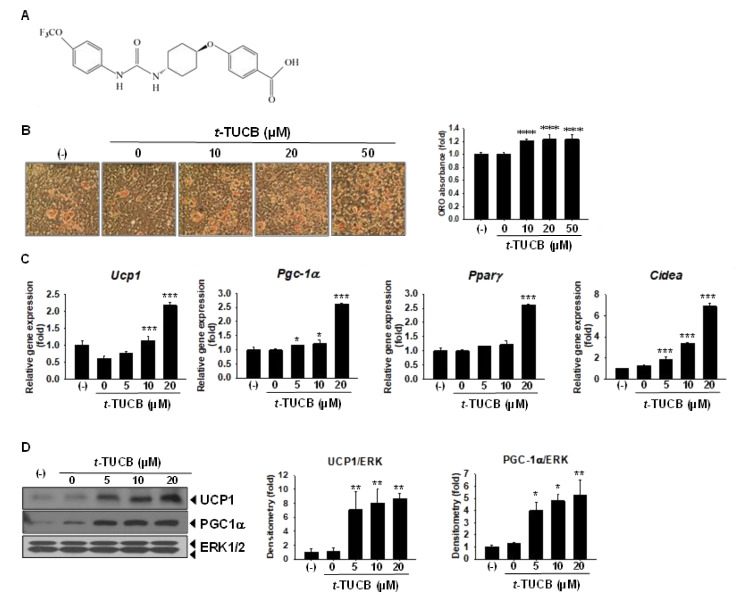
sEH inhibitor *t*-TUCB promotes murine brown adipogenesis in vitro. (**A**–**D**) Murine brown preadipocytes were differentiated in the presence of *t*-TUCB, as indicated for 6 days. (**A**) Chemical structure of *t*-TUCB. (**B**) Oil red O stained cell morphology and ORO absorbance. Data = Mean+SEM (n = 9) (**C**) Relative mRNA levels of brown marker gene *Ucp1*, *Pgc-1α*, *Pparγ*, and *Cidea*. mRNA expression was quantified relatively to the loading control *36b4*. (**D**) Protein expression of UCP1, PGC-1α, PPARγ, and the loading control ERK1/2. Bar graphs show normalized densitometry for UCP1/ERK1/2, PGC-1α/ERK1/2, and PPARγ/ERK1/2. Data = Mean+SEM (n = 3). *, **, ***, *p* < 0.05, *p* < 0.01, and *p* < 0.001 compared to the vehicle control (*t*-TUCB 0 samples), respectively.

Based on the sEH expression results, we hypothesized that sEH might play a role in brown adipogenesis in vitro, and sEH inhibition may modulate the process. *t*-TUCB (Figure 3A), a selective inhibitor of sEH [22], was chosen for the studies. *t*-TUCB significantly increased oil red O (ORO) stained differentiated brown adipocyte morphology and lipid accumulation, as assessed by the ORO absorbance (Figure 3B). *t*-TUCB significantly increased mRNA levels of brown marker genes *Ucp1*, *Pgc-1α*, and cell death-inducing DFFA like effector *A* (*Cidea*) (Figure 3C). *Pparγ* mRNA level was significantly increased by *t*-TUCB only at 20 µM (Figure 3C). Protein expression of UCP1 and PGC-1α were also increased by *t*-TUCB (Figure 3D). Interestingly, *t*-TUCB also increased *Ephx2* mRNA and sEH protein expression in a dose-dependent manner (Supplemental Figure S1), suggesting that there might be a feedback mechanism between *t*-TUCB and *Ephx2* gene expression. 

**Figure 4:**
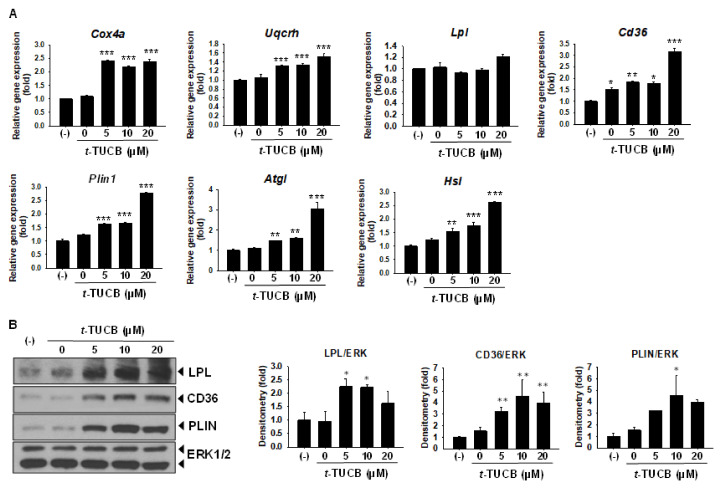
sEH inhibitor *t*-TUCB upregulates mitochondrial respiratory chain and lipid metabolic genes in murine brown adipocytes. Murine brown preadipocytes were differentiated in the presence of *t*-TUCB, as indicated for 6 days. (**A**) Relative mRNA levels of mitochondrial respiratory chain components (*Cox4a* and *Uqcrh*), fatty acid uptake genes (*Lpl* and *Cd36*), and lipolytic genes (*Plin1*, *Atgl*, and *Hsl*). mRNA expression was quantified relatively to the loading control *36b4*. (**B**) Protein expression of LPL, CD36, PLIN, and the loading control ERK1/2. Data = Mean+SEM (n = 3). *, **, ***, *p* < 0.05, *p* < 0.01, and *p* < 0.001 compared to *t*-TUCB 0 samples, respectively.

Moreover, we examined the effects of *t*-TUCB on mRNA expression of genes related to the brown adipocyte thermogenic function, i.e., mitochondrial respiratory chain and lipid metabolic genes. It is known when brown adipocytes are activated by cold or β adrenergic stimulation, lipid uptake and lipolysis are increased as fatty acids are not only used as fuel for oxidation but allosterically activate UCP1 [1]. *t*-TUCB significantly increased mRNA levels of mitochondrial cytochrome c oxidase subunit IV isoform (*Cox4a*, a subunit of respiratory chain Complex IV) and cytochrome b-c1 complex subunit 6 (*Uqcrh*, a subunit of respiratory chain Complex III) (Figure 4A). Of the two primary genes responsible for taking up fatty acids from the circulation into the brown adipocytes lipoprotein lipase (*Lpl)* and cluster of differentiation 36 (*Cd36)*, *t*-TUCB did not change *Lpl* mRNA levels but significantly increased *Cd36* mRNA levels (Figure 4A). *t*-TUCB also significantly increased mRNA levels of perilipin (*Plin1*), a lipid-coating protein that controls the access of lipases to the lipid droplet, and mRNA levels of adipose tissue triglyceride lipase (*Atgl*), and hormone-sensitive lipase (*Hsl*). *Atgl* and *Hsl* catalyze the removal of fatty acids from sn-1 and sn-2 position to generate diacylglycerol and monoacylglycerol, respectively. Protein expression of LPL, CD36, and PLIN were also increased by *t*-TUCB (Figure 4B). 

**Figure 5:**
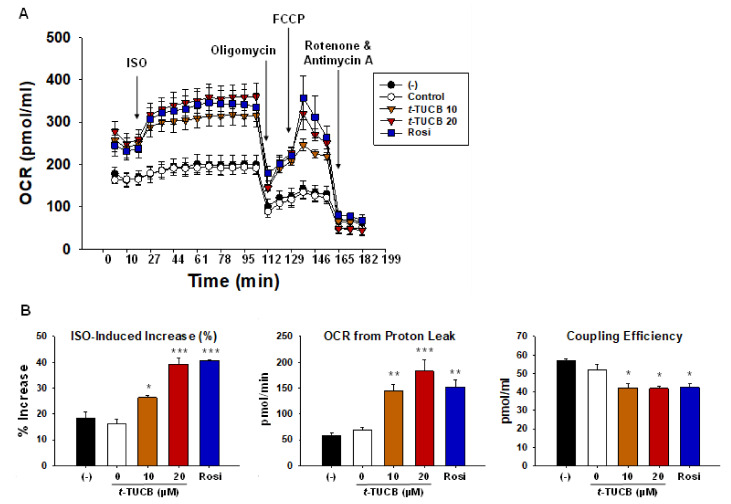
sEH inhibitor *t*-TUCB increases mitochondrial uncoupling in murine brown adipocytes. Murine brown preadipocytes were differentiated in the presence of *t*-TUCB or Rosi, as indicated for 6 days. Then brown adipocytes were reseeded onto a 24-well XF assay plate at 5.0 × 10^4^ cells per well. The next day, the cells were subjected to real-time measurements of OCR using an XF24 Extracellular Flux Analyzer. (**A**) OCR overtime during mitochondrial stress tests. (**B**) Isoproterenol (ISO)-induced OCR % increase, OCR from proton leak, and coupling efficiency. Data = Mean + SEM (n = 3). *, **, ***, *p* < 0.05, *p* < 0.01, and *p* < 0.001 compared to *t*-TUCB 0 samples, respectively.

To confirm whether *t*-TUCB increases mitochondrial respiration in murine brown adipocytes, cellular energetics were measured using an XF24 Extracellular Flux Analyzer (Figure 5). PPARγ has been recognized as a master regulator for both white and brown adipocyte differentiation [23] and rosiglitazone (Rosi), a synthetic ligand for PPARγ, was shown to promote brown adipocyte differentiation [24]; therefore, we included Rosi as a positive control in our assays. *t*-TUCB or Rosi-treated brown adipocytes had a higher basal oxygen consumption rate (OCR) compared to the controls (Figure 5A). Isoproterenol (ISO), a non-selective β adrenoreceptor agonist, significantly increased OCR in Rosi-treated cells (*p* < 0.001), compared to the controls (Figure 5B Left panel). ISO significantly increased OCR in the cells treated with *t*-TUCB at 10 and 20 µM (*p* < 0.05 and *p* < 0.001, respectively) (Figure 5B Left panel). Similar to Rosi, *t*-TUCB treated cells also had higher OCR produced by proton leak (i.e., mitochondrial uncoupling) (*p* < 0.01 and *p* < 0.001 for *t*-TUCB at 10 and 20 µM; *p* < 0.01 for Rosi) (Figure 5B Middle panel) and lower coupling efficiency (i.e., percentage of OCR for ATP production) (*p* < 0.05 for *t*-TUCB at 10 and 20 µM and for Rosi) (Figure 5B Right panel). In addition, *t*-TUCB treated cells had significantly higher OCR for ATP synthesis (*p* < 0.05 by *t*-TUCB at 20 µM) and no significant changes in maximal respiration compared to the controls (Supplemental Figure S2). 

### 2.3. t-TUCB Activates both PPARγ and PPARα

**Figure 6:**
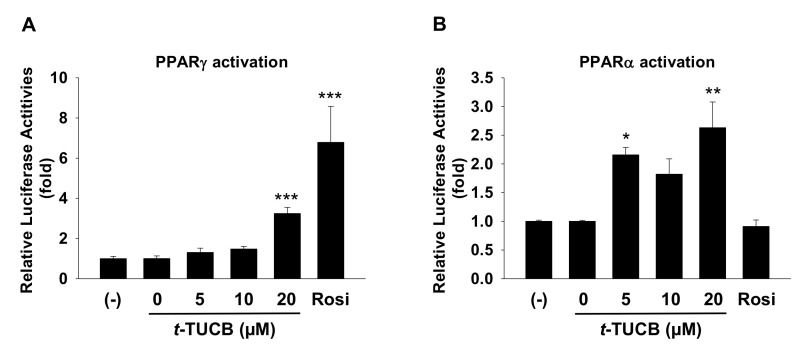
*t*-TUCB activates both PPARγ and PPARα in murine brown preadipocytes. Murine brown preadipocytes were transiently transfected with murine PPARγ (**A**) or PPARα (**B**) transactivation reporters and β-gal plasmid for 24 h. The cells were treated with *t*-TUCB (5, 10, 20 µM), Rosi, or the vehicle control for 18 h. Reporter gene assays were performed and normalized to β-gal activity. Relative luciferase activities were expressed as fold of the controls (set as 1). Data = Mean + SEM (n = 3). *, **, ***, *p* < 0.05, *p* < 0.01, and *p* < 0.001 compared to the controls, respectively.

To explore the molecular mechanisms by which the sEH inhibitor promotes murine brown differentiation, the abilities of *t*-TUCB to activate PPARγ and PPARα were tested using the respective transactivation reporter assays. *t*-TUCB at 20 µM significantly activated both PPARγ and PPARα (*p* < 0.001 for PPARγ and *p* < 0.01 for PPAR*α*, respectively) whereas *t*-TUCB at 5 µM only activated PPARα (*p* < 0.05) (Figure 6A,B). It is not known if *t*-TUCB acted by stabilizing EpFAs, as a PPAR agonist, or possibly both since *t*-TUCB was designed as a mimic of EET [25], which has been shown to have PPAR agonist activity [26].

### 2.4. t-TUCB Delivered via Mini Osmotic Pump Decreases Serum Triglycerides in Diet-Induced Obese Mice

**Figure 7:**
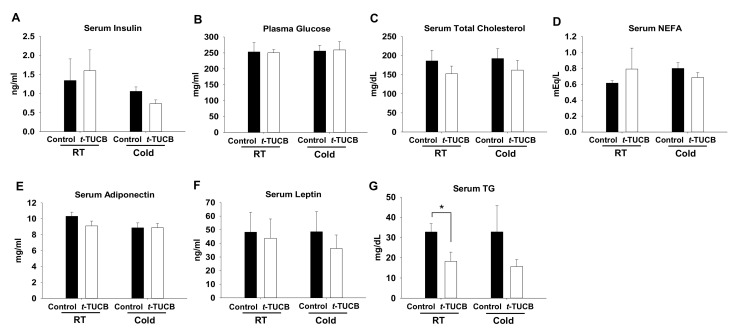
*t*-TUCB delivered via mini osmotic pump decreases serum triglycerides in diet-induced obese mice. C57BL6/J mice were fed a high-fat diet for 8 weeks, followed by *t*-TUCB (3 mg/kg/day) or the vehicle containing osmotic minipump implantation for 6 weeks as described. Serum insulin (**A**), plasma glucose (**B**), serum total cholesterol (**C**), NEFA (**D**), adiponectin (**E**), leptin (**F**), and TG (**G**) levels in the control and *t*-TUCB treated mice in the RT and cold condition. Data = Mean + SEM (n = 4–). *, *p* < 0.05 compared to the controls, respectively.

To confirm whether the effects of sEH inhibition by *t*-TUCB on brown adipogenesis and function can be beneficial in DIO in a mouse model, osmotic minipumps filled with *t*-TUCB to provide 3 mg/kg/day were implanted into the subcutaneous area on top of the iBAT of the diet-induced obese C57BL/6J male mice. The purpose for such delivery was to maximize the effects of *t*-TUCB on the iBAT while minimizing its systemic effects. After 6 weeks of treatment, *t*-TUCB did not change the body weight, food intake, fasting glucose levels, or individual fat pad mass, including iBAT (Supplemental Figure S3A–D). In addition, *t*-TUCB did not change glucose tolerance, insulin sensitivity, or cold tolerance (6 h at 4 °C) (Supplemental Figure S3E–G). To examine whether local delivery of *t*-TUCB can improve body metabolism, we performed blood biochemical analysis (Figure 7). *t*-TUCB did not change blood levels of insulin, glucose, total cholesterol, non-esterified fatty acid (NEFA), adiponectin, or leptin levels, but decreased serum triglyceride (TG) levels both in room temperature (RT) and cold condition (*p* < 0.05 in RT) (Figure 7).

### 2.5. t-TUCB Delivered via Mini Osmotic Pump Increases Protein Expression of Genes Involved in Lipid Metabolism in the iBAT of Diet-Induced Obese Mice

**Figure 8:**
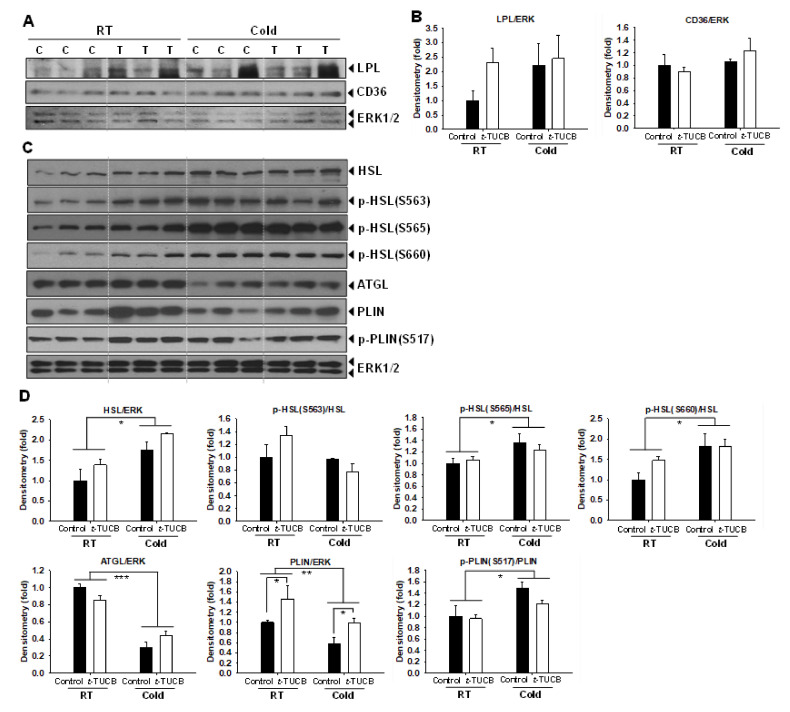
*t*-TUCB delivered via mini osmotic pump increases protein expression of genes involved in lipid metabolism in the interscapular brown adipose tissue of diet-induced obese mice. (**A**,**B**) Protein expression of LPL, CD36, and the loading control ERK in the iBAT of *t*-TUCB treated and the control mice, and their densitometry. (**C**,**D**) Protein expression of HSL, ATGL, PLIN, the phosphorylation of HSL and PLIN, and the loading control ERK in the iBAT of *t*-TUCB treated or the control mice, and their densitometry. Bar graphs show normalized densitometry for LPL/ERK1/2 and CD36/ERK1/2 in (**B**) HSL/ERK1/2, ATGL/ERK1/2, PLIN/ERK1/2, and p-HSL(S563)/HSL, p-HSL(S565)/HSL, p-HSL(S660)/HSL and p-PLIN(S517)/PLIN in (**D**). Data = Mean+SEM (n = 3). *, **, ***, *p* < 0.05, *p* < 0.01, and *p* < 0.001 compared to the controls, respectively.

Histological analysis of the iBAT slides showed that *t*-TUCB treated mice had less lipid accumulation caused by high-fat feeding than the control mice; however, the changes were not significant (Supplemental Figure S4A,B). Analysis of thermogenic protein expression in the iBAT revealed that *t*-TUCB treated mice had no significant increases in UCP1 and PGC1α protein expression in the iBAT compared with the controls both in RT and the cold condition (Supplemental Figure S4C). Since *t*-TUCB treated mice had significantly lower TG levels, we examined the protein expression of genes involved in fatty acid uptake from the circulation LPL and CD36 in the iBAT. *t*-TUCB treated mice had higher LPL protein expression; however, the changes did not reach statistical significance. There were no significant changes in CD36 expression in the iBAT in the *t*-TUCB treated mice compared to the controls (Figure 8A,B). 

We further analyze the expression and phosphorylation of the proteins involved in the iBAT lipolysis. The cold exposure significantly increased HSL protein abundance and phosphorylation of HSL at S565 and S660 (*p* < 0.05) but did not affect the phosphorylation of HSL at S563. *t*-TUCB treated mice had higher HSL protein abundance and higher phosphorylation of HSL at S563 and S660 at RT (Figure 8C,D); however, the changes did not reach statistical significance. Moreover, cold exposure decreased ATGL protein abundance (*p* < 0.01); however, *t*-TUCB treatment did not affect ATGL protein expression (Figure 8C,D). On the other hand, the cold exposure significantly decreased PLIN protein abundance (*p* < 0.01) but increased phosphorylation of PLIN at S517 (*p* < 0.05) (Figure 8C,D). *t*-TUCB treated mice had significantly higher PLIN expression in the iBAT both in RT and the cold condition (*p* < 0.05), but no change in the phosphorylation of PLIN at S517 compared to the controls in either RT or the cold conditions (Figure 8C,D). 

## 3. Discussion

Although questions remain on the composition and origin of BAT in adult humans [27,28], it is generally believed that BAT is a novel target to combat human obesity and associated metabolic disorders. Here, we show the sEH expression pattern in the murine and human brown adipogenesis in vitro and the WAT and BAT fat in the DIO mice. Moreover, the sEH inhibitor *t*-TUCB dose-dependently promoted brown adipogenesis and mitochondrial uncoupling. Furthermore, *t*-TUCB treatment via mini osmotic pump delivery decreased serum TG levels, which was accompanied by higher lipolytic protein expression, such as PLIN, in the iBAT. 

To our knowledge, the study is the first to report sEH expression pattern in cellular brown adipogenesis of both murine and human origins and the effects of sEH inhibition in the murine model. We found that sEH expression was increased during both murine and human brown adipocyte differentiation, which are in line with sEH expression patterns in 3T3-L1 white adipocyte differentiation [14]. Also, higher sEH activities were reported in the differentiated white adipocytes compared to the undifferentiated counterparts, as indicated by lower levels of total EETs and higher levels of their diol products DHET [29]. Our oxylipin analysis shows that some diol/epoxide ratios were significantly higher in the media from differentiated brown adipocytes than from the preadipocytes, which indicates higher sEH activities in the differentiated brown adipocytes, consistent with increased sEH protein expression. As many EpFAs, the substrates for sEH, are autocrine/paracrine lipid signaling molecules that play essential roles in various aspects of cell biology, including cell differentiation. For example, EETs, the type of EpFAs produced from AA, can bind and activate PPARγ [26]. PPARγ is a master regulator for both white and brown adipocyte differentiation [23]. Therefore, sEH may serve as a negative feedback for adipocyte differentiation by deactivating EETs during the process. However, cautions have to be taken in the interpretation of the oxylipin results. Several factors may influence the ratios besides sEH activity, including rapid metabolism, excretion of diols, and differential localization in cells. Additionally, epoxy fatty acids can be reincorporated into phospholipids or other esters, all of which may explain why not all ratios of diol/epoxide detected from the cell culture media were increased in our analysis. Future studies are warranted to investigate the mechanisms and the role of the increases in sEH activities in brown adipocytes as well as the fates of EpFAs and diols in these cells. 

In comparison to the effects of *t*-TUCB on brown adipocyte differentiation, additions of exogenous EETs suppressed white adipocyte differentiation from the mesenchymal stem cells [29]. Moreover, the addition of a different sEH inhibitor, 12-(3-adamantane-1-yl-ureido)-dodecanoic acid (AUDA), enhanced the suppressive effects of EETs [29]. Also, an EET analog was also shown to suppress the accumulation of large lipid droplets, a marker for adipocyte differentiation, in 3T3-L1 adipocytes [30]. However, EETs in the presence of AUDA were shown to activate PPARγ and increase mRNA expression of fatty acid binding protein 4 in 3T3-L1 preadipocytes, which is indicative of adipocyte differentiation [26]. These results suggest that the sEH inhibitor may have complex effects on white adipocyte vs. brown adipocyte differentiation. Moreover, significant upregulation of *Ephx2* mRNA levels were only found in the iBAT, but not in the eWAT and iWAT, which is consistent with a previous report [14]. Therefore, it appears that sEH may play differential roles in the BAT and WAT in DIO and sEH inhibitors may have differential effects on BAT and WAT development *in vivo*. BAT and various WAT depots are known to have differences in energy metabolism and physiological functions. BAT is responsible for UCP1 mediated thermogenesis, whereas WAT is responsible for energy storage. Among various WAT depots, iWAT are thought to be more prone to form beige adipocytes (i.e., UCP1 expressing brown-like adipocytes) capable of producing heat in response to cold challenge. Despite the drastic differences between the BAT and WAT, no significant differences were found between the DIO mice and the controls in *Ucp1* mRNA levels in the iBAT and iWAT, although in the eWAT the DIO mice had higher *Ucp1* mRNA levels than the controls. These findings raise the questions of whether sEH plays differential roles in brown vs. white adipogenesis and in the BAT vs. WAT in the development of diet-induced obesity. Further studies are needed to define the roles of sEH and the effects of sEH inhibition in various fat depots in diet-induced obesity. 

Based on the brown cell culture results, we have focused on the effects of sEH inhibition on the iBAT in our animal studies.To explore the mechanisms by which *t*-TUCB improved serum TG levels in diet-induced obese mice, we analyzed protein expression involved in TG uptake and breakdown in the iBAT. It has been reported that LPL and CD36, two genes primarily responsible for the hydrolysis of circulating triglyceride-rich lipoproteins and taking up fatty acids, respectively, were both upregulated in the BAT and required for increased TG clearance in response to the cold [31]. In addition, LPL expression was also increased by cold-induced lipokine 12,13-DiHOME in the BAT [32]. Injection of 12,13-DiHOME into obese B6 mice was shown to decrease serum TG without affecting body weight and glucose tolerance by increasing fatty acid uptake into the BAT [32]. Increased fatty acid uptake was attributed to increased translocation of fatty acid transporters, such as CD36, in brown adipocytes by the lipokine [32]. Due to limited tissue samples, we could not investigate the effects of *t*-TUCB on CD36 translocation. Future studies are needed to investigate the effects on *t*-TUCB on fatty acid uptake in brown adipocytes. It is worth noting that *t*-TUCB dose-dependently increased both mRNA and protein expression of CD36 and protein (but not mRNA) expression of LPL in vitro. Therefore, decreased serum TG levels by *t*-TUCB local delivery seems to be consistent with the published reports on BAT activation by the cold and the lipokine [31,32] and suggests that *t*-TUCB may enhance BAT activity, which in turn can promote TG clearance from circulation by increasing hydrolysis of TG and possibly fatty acid uptake into the brown adipocytes.

In adaptive thermogenesis, BAT responds to the β-adrenergic stimulation by increasing lipolysis to provide fatty acids as substrates for heat production via uncoupling. As the most abundant lipid-coating protein on mature lipid droplet (LD), *Plin1* is critical for norepinephrine (NE)-induced lipolysis in the BAT and thermal response to NE in vivo [33]. Moreover, transgenic mice with adipose-specific aP2 promoter/enhancer driven murine *Plin1* overexpression led to significant increases of mRNA expression of fatty acid oxidative genes in the BAT, but not in the WAT [34,35]. Furthermore, phosphorylation of murine PLIN sequence on serine 517 (equivalent to human PLIN serine 522) by activated protein kinase A (PKA) in response to cold exposure is essential for the ATGL activity [36]. ATGLcatalyzes the removal of sn-1 fatty acid from the stored TGs to produce diacylglycerol (DAG) [37]. DAG can be further hydrolyzed by HSL producing monoacylglycerol, which is further hydrolyzed by the monoacylglycerol lipase [38]. Phosphorylation of HSL on Ser 563, Ser 565, and Ser 660 have been shown to affect HSL-mediated lipolysis [39,40,41]. It is worth noting that *t*-TUCB dose-dependently increased mRNA and protein expression of *Plin1* and mRNA expression of *Atgl* and *Hsl* in murine brown adipocytes in vitro. Increases in lipolytic gene expression, together with increases in thermogenic UCP1 and PGC-1α expression, are consistent with increases in ISO-stimulated oxygen consumption and mitochondrial uncoupling in *t*-TUCB treated brown adipocytes in vitro. Together, our combined in vivo and in vitro results suggest that *t*-TUCB may promote BAT activity by increasing protein expression involved in lipolysis, leading to less lipid accumulation in the BAT and significantly lower serum TG levels in the diet-induced obesity. 

It should be noted that our studies cannot rule out the effects of *t*-TUCB on other tissues, such as subcutaneous white fat and liver, to improve lipid metabolism. Previous studies have shown sEH inhibition reduced inflammation [16], reduced ER stress [15], and restored autophagy [20] in the white adipose and liver, and improved the overall metabolic health in DIO of mice. In addition, it is worth noting that the BAT is partially activated at 22–23 °C (RT) and further activated at 4 °C (Cold) in our studies; however, there was minimal browning of iWAT and eWAT in the mice either at RT or at 4 °C for only 6 h (data not shown). Therefore, the observed decreases in TG levels in *t*-TUCB treated mice at RT are more likely due to the enhanced BAT activities rather than enhanced browning of white fat. Previous studies by others have also investigated this topic. It was reported that sEH genetic deficiency led to the reprogramming of white fat with increases in mitochondrial and thermogenic *Ucp1* mRNA expression [42]. Similarly, an EET analog was shown to induce UCP1 protein expression in 3T3-L1 adipocytes [30]. Therefore, it is possible that sEH inhibition could affect browning; however, our current study designs do not support the study of browning of the WAT. Future studies are needed to study the effects of *t*-TUCB on browning of WAT. 

In a preventive study, *t*-TUCB was systemically administered at ~1.67 mg/kg/day for 16 weeks starting at the same time with a high-fat diet; it increased BAT volume of *fat-1* mice, but not the WT controls [20]. Due to overexpression of an n-3 desaturase, *fat-1* mice had increased endogenous levels of n-3 PUFA and n-3 PUFA derived EpFAs. The *t*-TUCB administration further increased n-3 PUFA derived EpFAs in the *fat-1*-mice. In contrast, *t*-TUCB did not seem to affect the weight gain or total (white) fat volume in either *fat-1* or WT mice on the high-fat diet [20]. Our studies further add to the current understanding by demonstrating the effects of *t*-TUCB on brown adipogenesis in vitro and on improving TG levels in high fat diet-induced obese mice. The *t*-TUCB dose at 3 mg/kg/day we used has been demonstrated by other studies to achieve beneficial effects with minimal toxicity in mice [20,43]. Moreover, when administered at 3 mg/kg, the mice blood *t*-TUCB levels reached micromolar levels [44], which are within the concentration range we have used for the in vitro studies. On the other hand, our findings that *t*-TUCB at this dose for 6-weeks improved TG levels, but did not alter body weight in the obese mice seem to support the notion that enhanced BAT mass/activity may improve metabolisms in a relatively short period, but may require a longer duration of intervention to decrease body weights. For example, BAT transplantation improved high fat-induced glucose intolerance after 8 weeks of transplantation and only partially attenuated the body weight after 12 weeks of transplantation [45]. Together, these results suggest that sEH inhibition by *t*-TUCB may be a novel strategy for diet-induced obesity by promoting brown adipogenesis and BAT activities; however, a long duration of treatment or combination with EpFA-generating diets may be necessary to reduce the body weight gain and other aspects of metabolism in obesity. 

The mechanisms by which the sEH inhibitors promoted brown differentiation and improved BAT protein expression in the obese mice remain to be determined. We show that *t*-TUCB activated both PPARγ and PPARα in brown preadipocytes at the tested concentrations in vitro. PPARα and PPARγ are known to be expressed in the BAT [46,47]. Both PPARs modulate gene expression by binding to the PPAR response element(s) present in the target genes’ promoter in heterodimers with the retinoid X receptor. PPAR target genes include *Ucp1* [12,46], *Lpl* [46], and *Plin1* [47], which were found to be induced by *t*-TUCB in our studies. Herein, *t*-TUCB most likely functions either through the stabilization of EpFAs or by acting as a PPAR agonist. EETs, among the main EpFAs generated by CYPs from AA, were shown to bind and activate PPARs and induce target gene transcription [26]. Some of the biological effects of EETs and sEH inhibitors have been shown to be mediated through PPARγ in various disease models [26,48,49,50,51]. However, it is also possible that *t*-TUCB may mimic the effects of EpFAs by binding to PPARs directly as an agonist, as the structural component of urea present in *t*-TUCB has been used to mimic epoxides [25]. Future studies are needed to explore the mechanisms by which *t*-TUCB activates PPARs to modulate gene expression in brown adipocytes and BAT. 

In conclusion, although the exact molecular mechanisms are yet to be determined, the results presented herein show that sEH may be a negative regulator of brown adipogenesis; and sEH inhibition may be beneficial in improving BAT protein expression involved in lipid metabolism in diet-induced obesity. Further studies using the sEHI combined with EpFA generating diets for obesity treatment and prevention are warranted. 

## 4. Materials and Methods 

### 4.1. Reagents

sEH inhibitors *t*-TUCB was synthesized as described [52,53]. Anti-UCP1 antibody (Catalog# U6382) was purchased from Sigma Aldrich (St. Louis, MO, USA). Anti-PGC-1α antibody (Catalog# AB3242) was purchased from Millipore (Temecula, CA, USA). Antibody for phospho-perilipin (PLIN) (murine Ser 517) (Catalog# 4856) was from Vala Sciences (San Diego, CA, USA). LPL antibody (SC-373759) was purchased from Santa Cruz Biotechnology (Dallas, TX, USA). CD36 antibody (NB400-144) was purchased from Novus Biologicals (Centennial, CO, USA). Antibodies for phospho-HSL (Ser 563, Ser 565, and Ser 660), HSL, and PLIN were from Lipolysis Activation Antibody Sampler Kit (Catalog# 8334), Antibodies for ATGL (Catalog# 2439), PPARγ (Catalog# 2443) and ERK1/2 (Catalog# 4695) were obtained from Cell Signaling Technology (Danvers, MA). Anti-murine and anti-human sEH antibodies were generated and used, as described previously [54,55,56,57]. 

### 4.2. Cell Culture and Treatment

The murine brown preadipocyte cell line was a gift from Dr. Johannes Klein (University of Lubeck, Lubeck, Germany), which was generated from the interscapular brown fat of newborn C57BL/6J mice [58]. The cells were maintained in Dulbecco’s Modified Eagle’s medium (DMEM) supplemented with 20% fetal bovine serum (FBS) (Atlanta Biologicals, Flowery Branch, GA) in a humidified 37 °C and 5% CO_2_ incubator until they reached confluence (designated as day 0). The cells were induced to differentiate in differentiation media containing DMEM supplemented with 20% FBS, 1 nM T3, and 20 nM insulin every 2 days for 6 days. 

The human brown cell line is a gift from Dr. Yu-hua Tseng at Joslin Diabetes Center. Cells were cultured and differentiated, as previously described [59]. Briefly, human brown preadipocytes were grown in DMEM with 10% FBS until they reached confluence. The cells were then induced to differentiate in DMEM with 10% FBS supplemented with 0.5 µM human insulin, 2 nM T_3_, 33 µM biotin, 17 µM pantothenate, 0.1 µM dexamethasone, 0.5 mM 3-isobutyl-1-methylxanthine, and 30 µM indomethacin for 4 weeks. The media was replaced every 3 days. 

During the experiments, *t*-TUCB or the vehicle control (DMSO) were added at the indicated concentrations on day 0 (D0) of the differentiation and replaced with each change of media.

### 4.3. Analysis of Mitochondrial Respiration 

Murine brown preadipocytes were differentiated in the presence or absence of *t*-TUCB or Rosi for 6 days. Then the cells were collected and reseeded onto a 24-well XF assay plate at 5.0 × 10^4^ cells per well. The next day the cells were subjected to real-time measurements of oxygen consumption rate (OCR) and extracellular acidification rate (ECAR) using an XF24 Extracellular Flux Analyzer (Seahorse Biosciences, North Billerica, MA). First, basal measurements were taken, then the cells were stimulated with isoproterenol (1 µM) (via Port A injection) for 2 h followed by mitochondrial stress tests with injections via Port B-D sequentially in the following order: oligomycin (1 µM), carbonyl cyanide-ptrifluoromethoxyphenylhydrazone (FCCP; 1.5 µM), and antimycin A/rotenone (1 µM each). OCR and ECAR were automatically recorded by XF24 software v1.8 provided by the manufacturer. Isoproterenol-stimulated % increases of OCR rates were determined as the percentage of increases between the last measurements after ISO injection and the last basal measurements over the last basal measurements. Calculations of ISO-stimulated maximal OCR, OCR from ATP synthesis, OCR from proton leak, maximal respiration capacity, and coupling efficiency were performed according to the manufacturer’s instructions.

### 4.4. Reporter Gene Assays

Brown preadipocytes seeded on 48-well plates were transiently transfected with murine PPARγ or PPARα transactivation reporters and β-galactosidase expression plasmid with Lipofectamine 2000 transfection reagent and Plus reagent (Thermo Fisher Scientific, Carlsbad, CA, USA). PPARγ transactivation reporters consist of murine PPARγ ligand ligand-binding domain ligated to the Gal4 DNA binding domain (DBD) (mPPARγ-Gal4) and a reporter construct containing an upstream activating sequence (UAS)–linked luciferase, 4xUAS-TK-Luc (TK: thymidine kinase). PPARα transactivation reporters consist of murine PPARα ligand ligand-binding domain ligated to the DBD of the Gal4 DNA (mPPARα-Gal4) and 4xUAS-TK-Luc. Both reporters were gifts from Dr. Susanne Mandrup (University of Southern Denmark, Denmark) [60]. After 24 h, the cells were treated with sEH inhibitor *t*-TUCB or the vehicle control for 18 h. Cell lysates were prepared, and reporter luciferase and β-galactosidase activities were measured with GloMax Luminometer (Promega, Madison, WI, USA). Relative luciferase activity was normalized to β-galactosidase activity.

### 4.5. Oxylipin Analysis 

Oxylipin profiles were determined from the media collected from the cell culture of brown preadipocytes and differentiated adipocytes of murine or human origin. The media was collected after 12, 24, and 48 h after the incubation with the cells. Media (1.5 mL) of each sample set was spiked with 10 µL internal standard (a mixture of deuterated compounds) and extracted with solid-phase extraction cartridges (Oasis-HLB) (Waters, Milford, MA, USA). The extracted samples were then collected, dried, and reconstituted in 50 µL 200 nM 1-cyclohexyl-dodecanoic acid urea methanol solution. The analytes were then detected by LC-MS/MS according to the published method [61]. The raw data is provided as a separate Excel file. 

### 4.6. Animal Studies

All mice studies were approved by the Institutional Animal Care and Use Committee at the University of Tennessee, Knoxville (protocol 2587, approved on February 18, 2018). Mice were singly caged at 22–23 °C with 12 h light/dark shifts. The DIO mice and their chow-fed controls have been described [62,63]. Briefly, six week-old male C57BL/6J mice (n = 7 per group) (The Jackson Laboratory, Bar Harbor, ME, USA) were fed with either a high-fat diet (HF) (60% kcal from fat, D12492) (Research Diets Inc., New Brunswick, NJ, USA) or regular chow diet (RC) for 20 weeks before sacrificed at 26 weeks of age. Upon sacrifice, iBAT, epididymal and subcutaneous inguinal white adipose tissue (eWAT and iWAT) were excised and immediately snap-frozen in liquid nitrogen and stored at −80 °C until analysis.

For *t*-TUCB osmotic pump studies, male C57BL/6J mice (n = 10 per group) (The Jackson Laboratory) were purchased at 3 weeks old and were fed a high fat-diet diet (60% kcal from fat, D12492) (Research Diets) for 8 weeks to establish the obese condition. The mice were then surgically implanted with Alzet osmotic minipumps (model 2006) (DURECT Corporation, Cupertino, CA) filled *t*-TUCB into the subcutaneous compartment from an interscapular incision nearby the iBAT. *t*-TUCB was dissolved in a mixed solvent (25% DMSO in polyethylene glycol 400 (PEG400) as described [43] and delivered at 3 mg/kg/day for 6 weeks, which was the maximal delivery duration by design for the model. Insulin and glucose tolerance tests and indirect calorimetry were performed. Upon termination, four mice per group were randomly selected and subjected to cold tolerance tests. Immediately after the cold tests, these mice were euthanized as the cold exposed (Cold) mice after a whole blood collection by cardiac puncture under anesthesia. Various fat tissue, liver, and gastrocnemius muscle were collected and weighed. A small portion of the collected tissue was sampled for histological examination, and the rest was snap-frozen in liquid nitrogen then stored at −80 °C until analysis. The rest of the mice from each group were terminated the next day under room temperature (RT) following the same procedures. 

### 4.7. Blood Biochemical Analysis 

Plasma glucose was measured by Mouse Glucose Assay (Crystal Chem, Downers Grove, IL, USA). Serum insulin was measured using an Ultra Sensitive Mouse Insulin ELISA kit (Crystal Chem). Serum triglycerides (TG), non-esterified fatty acids (NEFA), and cholesterol were measured using respective tests from Wako Diagnostics (FUJIFILM Wako Diagnostics, Mountain View, CA, USA). Serum adiponectin and leptin were measured using the Mouse Quantikine ELISA kit for the respective analyte (R&D Systems, Minneapolis, MN, USA). All samples were analyzed according to the manufacturers’ instructions.

### 4.8. Western Blot Analysis

Total cell lysates were prepared using 1xRIPA lysis buffer (Cell Signaling, Danvers, MA, USA), and protein concentrations were determined using the BCA assay kit (Thermo Scientific, Waltham, MA, USA). Protein samples (20–30 µg per lane for cell samples and 1–50 µg per lane for tissue samples) were separated on a 10% SDS-PAGE and then transferred to polyvinylidene difluoride membranes (Bio-Rad, Hercules, CA, USA). The membranes were blocked in TBST buffer (20 mM Tris Base, 137 mM NaCl, and 0.1% Tween 20 (pH 7.4)) containing 5% nonfat milk. Membranes were then immunoblotted with the indicated primary antibodies against proteins of interest at 4 °C overnight at 1:1000 dilution (or 1:4000 for sEH antibody) followed by 1 h incubation with secondary antibodies conjugated with horseradish peroxidase at 1:4000 dilution. Bands of the protein of interest were visualized using SuperSignal West Pico Chemiluminescent Substrate (Thermo Scientific, Pittsburgh, PA). Densitometry of bands was quantified using Image Studio software (LI-COR Biosciences, Lincoln, NE, USA) or ChemiDoc XRS+ system with Image Lab software (Bio-Rad, Hercules, CA, USA). 

### 4.9. RNA Preparation and Quantitative Real-Time PCR Analysis

Total RNA was isolated using TRI reagent (Molecular Research Center, Cincinnati, OH, USA) according to the manufacturer’s instructions. Total RNA was quantified using the NanoDrop ND-1000 spectrophotometer (NanoDrop Technologies, Wilmington, DE, USA). Reverse transcription was carried out using a High Capacity cDNA Reverse Transcription kit (Thermo Scientific, Pittsburgh, PA, USA) according to the manufacturer’s instructions. mRNA expression of target genes and the housekeeping gene 36b4 (a ribosomal protein that is a component of the 60S subunit) was measured quantitatively using the PowerUp SYBR master mix (Applied Biosystems, Austin TX, USA). PCR reactions were run in a 96-well format using an ABI 7300HT instrument. Cycle conditions were 50 °C 2 min, 95 °C 10 min, and then 40 cycles of 95 °C for 15 s, 60 °C for 1 min. Relative gene expression was calculated using the 2-ΔΔCt method, after normalization to the indicated housekeeping gene. The primer sequences are shown in Supplemental Table S2. 

### 4.10. Statistical Analysis

The number of experiments and replicates are indicated in figure legends. Statistical analysis was performed using SigmaPlot 14.0 (Systat Software, San Jose, CA, USA) or Prism 8 (GraphPad Software, San Diego, CA, USA). One-way ANOVA with repeated measures, followed by the Student-Newman-Keuls method for multiple group-wise comparisons, was performed to determine the differences in group mean between the treatment groups and the control group for the cell culture studies. Two-way repeated-measures ANOVA was performed for oxylipin analysis of the cell media among 12, 24, and 48 h post-treatments and between pre- and differentiated states. Two-way ANOVA, followed by the same multi group-wise comparison, was performed to determine the differences between the treatment group and the control and between room temperature and cold condition for the animal studies. For the glucose insulin, and cold tolerance tests, areas under the curve (AUC) were calculated using Prism 8, and Student’s *t*-test was used to compare differences between the treatment and the control group. The level of significance was set at *p* < 0.05.

## Supplementary Materials

Supplementary materials can be found at https://www.mdpi.com/1422-0067/21/19/7039/s1.

## Abbreviations

AA	arachidonic acid
ALA	alpha-linolenic acid
ATGL	adipose triglyceride lipase
BAT	brown adipose tissue
β-gal	beta-galactosidase
DHA	docosahexaenoic acid
DHET	dihydroxyeicosatrienoic acid
DiHDPE	dihydroxydocosapentaenoic acid
DiHETE	dihydroxyeicosatetraenoic acid
DiHODE	dihydroxyoctadecadienoic acid
DiHOME	dihydroxyoctadecenoic acid
EET	epoxyeicosatrienoic acid
EPA	eicosapentaenoic acid
EpDPE	epoxydocosapentaenoic acid
EpETE	epoxyeicosatetraenoic acid
EpODE	epoxyoctadecadienoic acid
EpOME	epoxyoctadecenoic acid
ERK	extracellular signal-regulated kinases
GTT	glucose tolerance test
HSL	hormone sensitive lipase
ITT	insulin tolerance test
LA	linoleic acid
NEFA	non-esterified fatty acids
mEH	microsomal epoxide hydrolase
OCR	oxygen consumption rate
PGC-1α	peroxisome proliferator activator receptor coactivator 1-α
PLIN	perilipin
PPARα	peroxisome proliferator-activated receptor alpha
PPARγ	peroxisome proliferator-activated receptor gamma
PRDM16	PR domain containing 16
Rosi	Rosiglitazone
*t*-TUCB	trans-4-{4-[3-(4-trifluoromethoxy-phenyl)-ureido]-cyclohexyloxy-benzoic acid
sEH	soluble epoxide hydrolase
UCP1	uncoupling protein 1
WAT	white adipose tissue
