# Supplementary material for: Soluble Epoxide Hydrolase Inhibition by t-TUCB Promotes Brown Adipogenesis and Reduces Serum Triglycerides in Diet-Induced Obesity
Source: Int J Mol Sci. 2020 Sep 24;21(19):7039. doi: 10.3390/ijms21197039 (PMC7582898; doi:10.3390/ijms21197039)
Supplement: Supplementary file 1 [file ijms-21-07039-s001.zip › Supplemental Data Revised Based on PageProofs.docx]

**Supplemental Materials**

Soluble Epoxide Hydrolase Inhibition by *t*-TUCB Promotes Brown Adipogenesis and Reduces Serum Triglycerides in Diet-Induced Obesity by Haley Overby^1,^ *, Yang Yang^1,^ *, Xinyun Xu^1^, Katie Graham^1^, Kelsey Hildreth^1^, Sue Choi^1^, Debin Wan^3^, Christophe Morisseau^3^, Darryl C. Zeldin^4^, Bruce D. Hammock^3^, Shu Wang^5^, Ahmed Bettaieb^1, 2, #^, and Ling Zhao^1, #^

I. Supplemental Methods

1. Insulin and glucose tolerance test

For insulin tolerance tests, the mice were fasted for 6 hr before given insulin (Humulin) (Eli Lilly, Indianapolis, Indiana) at 0.75 U/kg of body weight through intraperitoneal injection. For glucose tolerance tests, the mice were fasted overnight (12-15 h) before orally gavaged with dextrose solution at 2 g/kg of bodyweight. Blood glucose levels were monitored and recorded at time 0, 15, 30, 60, 90, and 120 min after the administration of insulin or glucose with a handheld glucometer. These two tests were performed on all animals and at one week apart.

2. Cold tolerance test

Mice were randomly selected (n=4 per group) and singly housed in their home cage without the filter top and bedding at 4^O^C. Water and food were provided ad libitum. The mice body core temperatures were taken at time 0, 15, 30, 60, 90, 120, 150, and 180 min, 4 h, 5 h and 6 h using RET 3 ISO rectal probe for mice and a portable temperature monitor (Physitemp Instruments, Clifton, NJ).

3. Indirect calorimetry

Mice were placed singly in each chamber in the Comprehensive Lab Animal Monitoring System (CLAMS) (Columbus Instruments, Columbus, OH) under the same high-fat diet (60% kcal from fat) for 72 h. VO_2_, VCO_2_, body mass, and feedings were monitored and recorded according to the instructions by the manufacturer. Heat and the respiratory exchange ratio (RER) were calculated using the CLAMS data eXamination Tool (CLAX) statistical software provided by the manufacturer.

4. Lipid accumulation in the iBAT slices

Freshly isolated iBAT per mouse were sliced and fixed in 10% neutral buffered formalin before they were processed for hematoxylin and eosin staining at the University of Tennessee College of Veterinary Medicine Diagnostic Laboratory Service. 2-3 fields were taken per slide per mouse with a Nikon Eclipse E-600 microscopy. The pictures were analyzed by the Image J software.

II. Supplemental Figures and Tables

**Supplemental Fig S1 *t*-TUCB increases *Ephx2* mRNA and sEH protein expression in murine brown adipocytes.**

Murine brown preadipocytes were differentiated in the presence or absence of *t*-TUCB for 6 days. (A) Relative *Ephx2* mRNA levels; (B) Protein expression of sEH and the loading control ERK1/2. Bar graphs show normalized densitometry for sEH/ERK1/2. Data=Mean+SEM (n=3). **, ***, p<0.01, and p<0.001 compared to the vehicle control (*t*-TUCB 0 samples), respectively.

**Supplemental Fig S2 The effects of *t*-TUCB on OCR from ATP synthesis and maximal respiration in murine brown adipocytes.**

Murine brown preadipocytes were differentiated in the presence or absence of *t*-TUCB or Rosi for 6 days, followed by reseeded onto a 24-well XF assay plate at 5.0x10(4) per well. The next day the cells were subjected to real-time measurements of oxygen consumption rate (OCR) and extracellular acidification rate (ECAR) using XF24 Extracellular Flux Analyzer. OCR overtime during mitochondrial stress tests was recorded and calculated as described. (A) OCR from ATP synthesis; (B) Maximal Respiration. Data=Mean+SEM (n=3-4). One Way ANOVA. *, p<0.05 compared to the control.

**Supplemental Fig S3 The effects of *t*-TUCB minipump delivery on bodyweights, fasting glucose, fat pad weights, glucose and insulin tolerance, and cold tolerance in obese C57BL6/J mice.**

C57BL6/J mice were fed a high-fat diet for 8 weeks, followed by *t*-TUCB (3 mg/kg/day) (open bar or circle) or the vehicle (solid bar or circle) containing osmotic minipump implantation for 6 weeks. Bodyweight, food intakes, fasting glucose were recorded, and glucose and insulin and cold tolerance tests were performed as described. (A) Changes of bodyweight after the mini pump implantation; (B) Average food intake per day after the mini pump implantation; (C) Fasting glucose levels after 6 weeks of mini pump implantation; (D) Fat pad weights at termination; (E) Oral glucose tolerance test and areas under the curve; (F) Insulin tolerance test and areas under the curve; (G) Cold tolerance test and areas under the curve. Data=Mean+SEM (n=9-10 for A-F and n=4 for G). No significant differences were detected between the treated and the control group (Student’s t-test).

**Supplemental Fig S4 The effects of *t*-TUCB minipump delivery on lipid accumulation and UCP1 and PGC1α protein expression in the iBAT of obese C57BL6/J mice.**

After termination, the iBAT tissue slides from mice were stained with Hemotoxylin and Eosin (H&E) (A) and % area occupied by lipid from 2-3 fields per slide per mouse (n=9-10) were measured using Image J software as described and graphed in (B). (C) Protein expression of UCP1 and PGC1α in the BAT of *t*-TUCB treated or the control mice. Bar graphs show normalized densitometry for UCP1/ERK and PGC1α/ERK. Data=Mean+SEM (n=3). No significant differences were detected between the groups.

|  |  | **Murine Brown** | | | **Human Brown** | | |
| --- | --- | --- | --- | --- | --- | --- | --- |
|  |  | **Pre** | **Diff** | **P-value** | **Pre** | **Diff** | **P-value** |
| **AA Metabolites** | | | | | | | |
| 5,6-DHET/5,6-EET | 12h | 0.092±0012 | 0.097±0.022 | ns | 0.242±0.014 | 0.348±0.153 | ns |
|  | 24h | 0.149±0.015 | 0.059±0.013 | ns | 0.429±0.138 | 0.155±0.042 | ns |
|  | 48h | 0.163±0.039 | 0.050±0.013 | ns | 0.684±0.406 | 0.173±0.029 | ns |
| 8,9-DHET/8,9-EET | 12h | 0.224±0.040 | 0.360±0.053 | ns | 0.213±0.020 | 0.273±0.052 | ns |
|  | 24h | 0.284±0.029 | 0.334±0.056 | ns | 0.295±0.066 | 0.176±0.040 | ns |
|  | 48h | 0.237±0.043 | 0.346±0.050 | ns | 0.214±0.053 | 0.354±0.160 | ns |
| 11,12-DHET/11,12-EET | 12h | 1.10±0.421 | 0.861±0.046 | ns | 0.749±0.067 | 0.415±0.087 | ns |
|  | 24h | 1.18±0.143 | 0.097±0.282 | ns | 0.822±0.028 | 1.66±0.930 | ns |
|  | 48h | 0.925±0.168 | 0.966±0.219 | ns | 1.51±0.163 | 0.625±0.054 | ns |
| 14,15-DHET/14,15-EET | 12h | 2.05±0.295^a^ | 2.41±0.285 | ns | 1.32±0.135^a^ | 2.39±0.314 | ns |
|  | 24h | 3.42±0.436^a,b^ | 2.34±0.612 | ns | 2.16±0.140^a^ | 2.02±0.184 | ns |
|  | 48h | 4.41±0.941^b^ | 2.65±0.629 | P<0.05 | 5.06±0.966^b^ | 1.72±0.187 | P<0.001 |
| **LA Metabolites** | | | | | | | |
| 9,10-DiHOME/9,10-EpOME | 12h | 2.69±0.490 | 5.93±0.527^a^ | P<0.05 | 4.01±0.257^a^ | 0.218± 0.029 | P<0.001 |
|  | 24h | 2.42±0.323 | 2.74±0.649^b^ | ns | 1.89±0.278^b^ | 0.706±0.163 | P<0.001 |
|  | 48h | 0.549±0.058 | 0.874±0.091^b^ | ns | 0.471±0.098^c^ | 0.716±0.042 | ns |
| 12,13-DiHOME/12,13-EpOME | 12h | 0.910±0.072 | 2.04±0.160 | P<0.05 | 2.36±0.094 | 0.339±0.010 | P<0.01 |
|  | 24h | 1.16±0.038 | 2.12±0.550 | ns | 2.11±0.100 | 1.20±0.400 | P<0.05 |
|  | 48h | 1.17±0.109 | 1.36±0.120 | ns | 2.30±0.307 | 1.02±0.098 | P<0.01 |
| **ALA Metabolites** | | | | | | | |
| 9,10-DiHODE/9,10-EpODE | 12h | 5.98±1.240 | 7.75±1.234^a^ | ns | 6.07±0.911^a^ | 0.598±0.288 | P<0.05 |
|  | 24h | 4.70±0.968 | 4.32±1.590^a^,^b^ | ns | 3.34±1.310^b^ | 1.71±0.672 | ns |
|  | 48h | 1.25±0.087 | 1.52±0.454^b^ | ns | 1.39±0.593^b^ | 1.95±0.224 | ns |
| 12,13-DiHODE/12,13-EpODE | 12h | 2.33±0.426 | 2.68±0.330^a^ | ns | 2.34±0.285 | 2.98±1.88 | ns |
|  | 24h | 2.53±0.309 | 9.86±3.14^b^ | P<0.05 | 3.35±0.235 | 2.05±0.351 | ns |
|  | 48h | 2.14±0.216 | 3.63±1.08^a^ | ns | 2.96±0.801 | 4.74±0.643 | ns |
| 15,16-DiHODE/15,16-EpODE | 12h | 7.72±1.48 | 13.2±3.19 | ns | 6.88±0.303 | 16.8±8.81 | ns |
|  | 24h | 10.0±0.687 | 24.2±4.03 | P<0.05 | 8.24±0.917 | 20.9±9.73 | ns |
|  | 48h | 11.9±1.65 | 20.4±3.89 | ns | 14.1±2.37 | 17.7±4.25 | ns |
| **EPA Metabolites** | | | | | | | |
| 17,18-DiHETE/17,18-EpETE | 12h | 44.8±8.62 | 52.5±17.2 | ns | 14.2±1.44 | 40.8±7.59 | ns |
|  | 24h | 42.3±7.23 | 64.5±22.9 | ns | 15.4±5.03 | 18.9±7.45 | ns |
|  | 48h | 74.5±14.0 | 62.4±25.1 | ns | 37.8±12.5 | 23.8±1.46 | ns |
| **DHA Metabolites** | | | | | | | |
| 7,8-DiHDPE/7,8-EpDPE | 12h | 0.0170±0.00364^a^ | 0.0243±0.00246 | P<0.05 | 0.0318±0.0067 | 0.0699±0.0238 | P<0.05 |
|  | 24h | 0.0447±0.00677^b^ | 0.0349±0.00869 | ns | 0.0287±0.0104 | 0.139±0.0416 | P<0.05 |
|  | 48h | 0.0528±0.0175^b^ | 0.0262±0.00244 | ns | 0.0712±0.0131 | 0.0819±0.0197 | P<0.05 |
| 10,11-DiHDPE/10,11-EpDPE | 12h | 3.11±0.516^a,b^ | 2.35±0.335 | ns | 0.921±0.101^a^ | 0.720±0.0231 | P<0.05 |
|  | 24h | 3.96±0.0553^a^ | 1.85±0.357 | P<0.01 | 1.28±0.036^a^ | 0.796±0.0100 | P<0.05 |
|  | 48h | 2.35±0.424^b^ | 1.72±0.127 | ns | 1.95±0.188^b^ | 1.14±0.144 | P<0.05 |
| 13,14-DiHDPE/13,14-EpDPE | 12h | 1.02±0.248^a^ | 1.05±0.151 | ns | 0.472±0.0493 | 2.88±1.05 | ns |
|  | 24h | 1.74±0.240^a,b^ | 1.42±0.330 | ns | 0.691±0.0591 | 0.949±0.103 | ns |
|  | 48h | 3.22±0.944^b^ | 1.57±0.013 | ns | 1.72±0.386 | 1.16±0.139 | ns |
| 16,17-DiHDPE/16,17-EpDPE | 12h | 1.97±0.165^a^ | 1.62±0.313 | ns | 0.898±0.094 | 2.55±0.508 | ns |
|  | 24h | 4.35±0.570^a,b^ | 2.18±0.415 | P<0.05 | 1.09±0.0231 | 1.79±0.101 | ns |
|  | 48h | 5.11±0.932^b^ | 2.52±0.405 | P<0.05 | 4.31±1.02 | 2.34±0.346 | ns |
| 19,20-DiHDPE/19,20-EpDPE | 12h | 1.98±0.104^a^ | 2.50±0.049^a^ | ns | 0.954±0.088^a^ | 6.74±0.566 | P<0.01 |
|  | 24h | 6.11±0.369^a^ | 6.10±0.894^a,b^ | ns | 2.24±0.110^a^ | 4.63±0.422 | P<0.05 |
|  | 48h | 16.6±1.94^b^ | 9.30±1.68^b^ | P<0.01 | 6.92±0.0439^b^ | 0.716±0.042 | P<0.05 |

**Table S1.** Oxylipin analysis from the cell culture media collected from brown preadipocytes (Pre) and differentiated adipocytes (Diff) of murine or human origin. Ratios of detectable diols to their corresponding epoxy fatty acids as markers for sEH activity were calculated. Data= Mean±SEM (n=3). Two-way repeated-measures ANOVA was performed. P values are shown between Diff vs Pre samples of the same time point. ns, no significant difference. Different letters (a-c) indicate significant differences among 12, 24, and 48 h time points within the group.

Pre, preadipocyte; Diff, differentiated adipocyte; AA, arachidonic acid; LA, linoleic acid; ALA, alpha-linolenic acid; EPA, eicosapentaenoic acid; DHA, docosahexaenoic acid; DHET, dihydroxyeicosatrienoic acid; EET, epoxyeicosatrienoic acid; DiHOME, dihydroxyoctadecenoic acid; EpOME, epoxyoctadecenoic acid; DiHODE, dihydroxyoctadecadienoic acid; EpODE, epoxyoctadecadienoic acid; DiHETE, dihydroxyeicosatetraenoic acid; EpETE, epoxyeicosatetraenoic acid; DiHDPE, dihydroxydocosapentaenoic acid; EpDPE, epoxydocosapentaenoic acid.

| **Gene name** | **Primer sequences** | |
| --- | --- | --- |
| mouse *36b4* | Forward | GCTTCGTGTTCACCAAGGAGGA |
|  | Reverse | GTCCTAGACCAGTGTTCTGAGC |
| mouse *Ucp1* | Forward | GCTTTGCCTCACTCAGGATTGG |
|  | Reverse | CCAATGAACACTGCCACACCTC |
| mouse *Pgc-1α* | Forward | GAATCAAGCCACTACAGACACCG |
|  | Reverse | CATCCCTCTTGAGCCTTTCGTG |
| mouse *Pparγ* | Forward | GTACTGTCGGTTTCAGAAGTGCC |
|  | Reverse | ATCTCCGCCAACAGCTTCTCCT |
| mouse *Prdm16* | Forward | ATCCACAGCACGGTGAAGCCAT |
|  | Reverse | ACATCTGCCCACAGTCCTTGCA |
| mouse *Ephx2* | Forward | CCCTCAAGCAGTGTTCATTGGC |
|  | Reverse | ATCTGGTGGCATAAACGGCGTG |
| human *36B4* | Forward | TGGTCATCCAGCAGGTGTTCGA |
|  | Reverse | ACAGACACTGGCAACATTGCGG |
| human *UCP1* | Forward | AGTTCCTCACCGCAGGGAAAGA |
|  | Reverse | GTAGCGAGGTTTGATTCCGTGG |
| human *PGC-1α* | Forward | AGTGGTGCAGTGACCAATCA |
|  | Reverse | CTGCTAGCAAGTTTGCCTCA |
| human *PPARγ* | Forward | AGCCTGCGAAAGCCTTTTGGTG |
|  | Reverse | GGCTTCACATTCAGCAAACCTGG |
| human *PRDM16* | Forward | CAGCCAATCTCACCAGACACCT |
|  | Reverse | GTGGCACTTGAAAGGCTTCTCC |
| human *EPHX2* | Forward | AGCCTCTTCAGAGCAAGCGATG |
|  | Reverse | GGATTTCCTCCTCAGTGACCATC |
| **Table S2.**  Primer Sequences for semi-quantitative RT-PCR | | |
