# Supplementary material for: Soluble Epoxide Hydrolase Inhibition by t-TUCB Promotes Brown Adipogenesis and Reduces Serum Triglycerides in Diet-Induced Obesity
Source: Int J Mol Sci. 2020 Sep 24;21(19):7039. doi: 10.3390/ijms21197039 (PMC7582898; doi:10.3390/ijms21197039)
Supplement: Supplementary file 1 [file ijms-21-07039-s001.zip › Supplemental Table S2 oxylipin analysis (absolute) 5 7 20.pdf]

Media sample	Unit:nmol/L								
	**ARA**								
		CYPs				SEH			
Group Info.	Sample ID	14,15-EET	11,12-EET	8,9-EET	5,6-EET	14,15-DHET	11,12-DHET	8,9-DHET	5,6-DHET
Murine Brown Pre-adipocytes	12h sample 1	0.081	0.060	0.173	0.827	0.213	0.117	0.050	0.060
	12h sample 2	0.123	0.174	0.240	0.800	0.208	0.122	0.056	0.073
	12h sample 3	0.117	0.193	0.291	0.773	0.212	0.126	0.044	0.088
	Ave	0.107	0.142	0.235	0.800	0.211	0.121	0.050	0.074
	SE	0.013	0.041	0.034	0.015	0.002	0.003	0.003	0.008
	24h sample 1	0.077	0.116	0.149	0.400	0.276	0.119	0.044	0.048
	24h sample 2	0.105	0.106	0.136	0.353	0.273	0.111	0.045	0.055
	24h sample 3	0.063	0.080	0.131	0.284	0.256	0.118	0.030	0.049
	Ave	0.082	0.101	0.139	0.346	0.269	0.116	0.040	0.051
	SE	0.012	0.011	0.005	0.034	0.006	0.002	0.005	0.002
	48h sample 1	0.066	0.092	0.073	0.254	0.377	0.109	0.023	0.035
	48h sample 2	0.075	0.109	0.113	0.201	0.367	0.107	0.026	0.048
	48h sample 3	0.136	0.195	0.163	0.473	0.353	0.119	0.027	0.052
	Ave	0.092	0.132	0.116	0.310	0.366	0.112	0.025	0.045
	SE	0.022	0.032	0.026	0.083	0.007	0.004	0.001	0.005
Murine Differentiated Brown Adipocytes	12h sample 1	0.080	0.178	0.268	0.820	0.237	0.140	0.080	0.075
	12h sample 2	0.108	0.163	0.201	1.283	0.223	0.139	0.094	0.080
	12h sample 3	0.105	0.163	0.249	0.783	0.230	0.154	0.078	0.108
	Ave	0.098	0.168	0.239	0.962	0.230	0.144	0.084	0.087
	SE	0.009	0.005	0.020	0.161	0.004	0.005	0.005	0.010
	24h sample 1	0.068	0.104	0.150	0.693	0.236	0.156	0.055	0.034
	24h sample 2	0.105	0.182	0.195	0.740	0.232	0.157	0.080	0.062
	24h sample 3	0.156	0.265	0.231	1.183	0.210	0.144	0.052	0.052
	Ave	0.110	0.184	0.192	0.872	0.226	0.153	0.062	0.049
	SE	0.025	0.047	0.023	0.156	0.008	0.004	0.009	0.008
	48h sample 1	0.092	0.387	0.343	1.183	0.353	0.229	0.088	0.066
	48h sample 2	0.112	0.152	0.194	0.803	0.262	0.206	0.068	0.055
	48h sample 3	0.133	0.186	0.181	0.973	0.231	0.178	0.078	0.024
	Ave	0.112	0.242	0.239	0.987	0.282	0.204	0.078	0.049
	SE	0.012	0.073	0.052	0.110	0.037	0.015	0.006	0.013
Human Brown Pre-adipocytes	12h sample 1	0.073	0.144	0.202	0.587	0.112	0.089	0.050	0.154
	12h sample 2	0.089	0.084	0.261	0.580	0.095	0.070	0.046	0.125
	12h sample 3	0.078	0.105	0.245	0.647	0.107	0.083	0.053	0.160
	Ave	0.080	0.111	0.236	0.604	0.105	0.081	0.050	0.146
	SE	0.005	0.018	0.017	0.021	0.005	0.006	0.002	0.011
	24h sample 1	0.059	0.097	0.142	0.203	0.126	0.076	0.032	0.081
	24h sample 2	0.057	0.078	0.142	0.337	0.109	0.068	0.033	0.069
	24h sample 3	0.052	0.100	0.108	0.140	0.126	0.081	0.046	0.096
	Ave	0.056	0.092	0.131	0.227	0.120	0.075	0.037	0.082
	SE	0.002	0.007	0.011	0.058	0.006	0.004	0.004	0.008
	48h sample 1	0.038	0.057	0.079	0.113	0.147	0.069	0.014	0.036
	48h sample 2	0.036	0.030	0.066	0.020	0.154	0.048	0.021	0.030
	48h sample 3	0.019	0.035	0.062	0.091	0.131	0.061	0.009	0.022
	Ave	0.031	0.041	0.069	0.075	0.144	0.059	0.015	0.029
	SE	0.006	0.008	0.005	0.028	0.007	0.006	0.004	0.004
Human Differentiated Brown Adipocytes	12h sample 1	0.047	0.131	0.080	0.154	0.095	0.038	0.025	0.039
	12h sample 2	0.044	0.061	0.094	0.056	0.095	0.035	0.016	0.036
	12h sample 3	0.027	0.045	0.054	0.174	0.081	0.017	0.018	0.025
	Ave	0.039	0.079	0.076	0.128	0.090	0.030	0.020	0.033
	SE	0.006	0.026	0.012	0.037	0.005	0.007	0.003	0.004
	24h sample 1	0.051	0.020	0.097	0.198	0.103	0.071	0.009	0.019
	24h sample 2	0.056	0.155	0.107	0.240	0.131	0.074	0.023	0.057
	24h sample 3	0.078	0.077	0.086	0.273	0.133	0.078	0.018	0.036
	Ave	0.062	0.084	0.097	0.237	0.122	0.075	0.017	0.037
	SE	0.008	0.039	0.006	0.022	0.010	0.002	0.004	0.011
	48h sample 1	0.084	0.143	0.137	0.224	0.115	0.075	0.030	0.041
	48h sample 2	0.062	0.086	0.107	0.190	0.112	0.061	0.018	0.041
	48h sample 3	0.053	0.100	0.079	0.297	0.107	0.065	0.053	0.035
	Ave	0.067	0.110	0.108	0.237	0.111	0.067	0.034	0.039
	SE	0.009	0.017	0.017	0.032	0.002	0.004	0.010	0.002

Media sample	Unit:nmol/L				
	**LA**				
		CYPs		SEH	
Group Info.	Sample ID	12,13-EpOME	9,10-EpOME	12,13-DiHOME	9,10-DiHOME
Murine Brown Pre-adipocytes	12h sample 1	2.007	0.533	2.103	1.950
	12h sample 2	2.460	0.807	2.153	1.897
	12h sample 3	2.713	0.943	2.190	1.947
	Ave	2.393	0.761	2.149	1.931
	SE	0.207	0.121	0.025	0.017
	24h sample 1	1.780	0.460	1.980	0.950
	24h sample 2	1.683	0.423	1.920	0.900
	24h sample 3	1.607	0.311	1.987	0.953
	Ave	1.690	0.398	1.962	0.934
	SE	0.050	0.045	0.021	0.017
	48h sample 1	0.933	0.340	1.230	0.204
	48h sample 2	0.963	0.383	1.200	0.235
	48h sample 3	1.320	0.580	1.267	0.252
	Ave	1.072	0.434	1.232	0.230
	SE	0.124	0.074	0.019	0.014
Murine Differentiated Brown Adipocytes	12h sample 1	1.053	0.370	2.037	1.807
	12h sample 2	0.953	0.271	1.750	1.770
	12h sample 3	0.870	0.316	2.050	2.017
	Ave	0.959	0.319	1.946	1.864
	SE	0.053	0.029	0.098	0.077
	24h sample 1	0.315	0.146	0.993	0.570
	24h sample 2	0.540	0.272	1.053	0.723
	24h sample 3	0.760	0.393	0.963	0.650
	Ave	0.538	0.271	1.003	0.648
	SE	0.128	0.071	0.026	0.044
	48h sample 1	0.787	0.503	0.880	0.383
	48h sample 2	0.460	0.326	0.673	0.343
	48h sample 3	0.353	0.244	0.527	0.197
	Ave	0.533	0.358	0.693	0.308
	SE	0.130	0.077	0.102	0.057
Human Brown Pre-adipocytes	12h sample 1	0.820	0.383	1.823	1.347
	12h sample 2	0.720	0.287	1.673	1.193
	12h sample 3	0.693	0.291	1.763	1.273
	Ave	0.744	0.321	1.753	1.271
	SE	0.039	0.031	0.044	0.044
	24h sample 1	0.723	0.257	1.627	0.610
	24h sample 2	0.727	0.393	1.393	0.553
	24h sample 3	0.727	0.320	1.577	0.610
	Ave	0.726	0.324	1.532	0.591
	SE	0.001	0.039	0.071	0.019
	48h sample 1	0.617	0.331	1.040	0.102
	48h sample 2	0.350	0.193	0.907	0.089
	48h sample 3	0.324	0.128	0.850	0.083
	Ave	0.430	0.217	0.932	0.091
	SE	0.094	0.060	0.056	0.006
Human Differentiated Brown Adipocytes	12h sample 1	0.560	0.493	0.180	0.087
	12h sample 2	0.337	0.299	0.120	0.082
	12h sample 3	0.257	0.174	0.087	0.036
	Ave	0.385	0.322	0.129	0.068
	SE	0.091	0.093	0.027	0.016
	24h sample 1	0.150	0.088	0.299	0.091
	24h sample 2	0.453	0.327	0.357	0.161
	24h sample 3	0.460	0.296	0.370	0.178
	Ave	0.354	0.237	0.342	0.143
	SE	0.102	0.075	0.022	0.027
	48h sample 1	0.557	0.357	0.463	0.227
	48h sample 2	0.360	0.215	0.383	0.168
	48h sample 3	0.330	0.218	0.383	0.159
	Ave	0.415	0.263	0.410	0.185
	SE	0.071	0.047	0.027	0.021

Media sample

Unit:nmol/L

	**ALA**						
		CYPs			SEH		
Group Info.	Sample ID	15(16)-EpODE	9(10)-EpODE	12(13)-EpODE	15,16-DiHODE	9,10-DiHODE	12,13-DiHODE
Murine Brown Pre-adipocytes	12h sample 1	0.160	0.025	0.019	1.570	0.205	0.058
	12h sample 2	0.177	0.038	0.034	1.507	0.221	0.083
	12h sample 3	0.252	0.042	0.038	1.227	0.166	0.059
	Ave	0.197	0.035	0.031	1.434	0.197	0.067
	SE	0.028	0.005	0.006	0.105	0.016	0.008
	24h sample 1	0.184	0.042	0.033	1.787	0.153	0.064
	24h sample 2	0.141	0.020	0.031	1.600	0.134	0.087
	24h sample 3	0.160	0.025	0.017	1.440	0.096	0.049
	Ave	0.162	0.029	0.027	1.609	0.127	0.067
	SE	0.012	0.007	0.005	0.100	0.017	0.011
	48h sample 1	0.084	0.032	0.022	1.157	0.035	0.038
	48h sample 2	0.080	0.023	0.018	1.060	0.032	0.040
	48h sample 3	0.142	0.041	0.020	1.217	0.051	0.050
	Ave	0.102	0.032	0.020	1.144	0.039	0.043
	SE	0.020	0.005	0.001	0.046	0.006	0.004
Murine Differentiated Brown Adipocytes	12h sample 1	0.131	0.021	0.016	1.380	0.133	0.052
	12h sample 2	0.084	0.021	0.026	1.640	0.143	0.070
	12h sample 3	0.161	0.015	0.027	1.520	0.153	0.056
	Ave	0.125	0.019	0.023	1.513	0.143	0.059
	SE	0.022	0.002	0.003	0.075	0.006	0.006
	24h sample 1	0.055	0.008	0.004	1.763	0.061	0.051
	24h sample 2	0.070	0.023	0.003	1.537	0.069	0.045
	24h sample 3	0.102	0.038	0.028	1.900	0.093	0.100
	Ave	0.076	0.023	0.012	1.733	0.074	0.065
	SE	0.014	0.009	0.008	0.106	0.010	0.018
	48h sample 1	0.124	0.053	0.010	2.150	0.047	0.048
	48h sample 2	0.102	0.024	0.018	1.613	0.057	0.080
	48h sample 3	0.050	0.014	0.014	1.417	0.018	0.021
	Ave	0.092	0.031	0.014	1.727	0.041	0.049
	SE	0.022	0.012	0.003	0.219	0.012	0.017
Human Brown Pre-adipocytes	12h sample 1	0.147	0.020	0.029	0.930	0.142	0.055
	12h sample 2	0.122	0.022	0.023	0.847	0.093	0.053
	12h sample 3	0.136	0.020	0.018	1.000	0.136	0.051
	Ave	0.135	0.021	0.023	0.926	0.124	0.053
	SE	0.007	0.001	0.003	0.044	0.015	0.001
	24h sample 1	0.096	0.015	0.016	0.970	0.090	0.061
	24h sample 2	0.109	0.040	0.019	0.800	0.066	0.056
	24h sample 3	0.124	0.034	0.024	0.900	0.083	0.078
	Ave	0.110	0.030	0.020	0.890	0.079	0.065
	SE	0.008	0.008	0.002	0.049	0.007	0.007
	48h sample 1	0.083	0.021	0.021	0.850	0.052	0.030
	48h sample 2	0.055	0.017	0.004	0.767	0.008	0.018
	48h sample 3	0.048	0.017	0.012	0.877	0.021	0.041
	Ave	0.062	0.018	0.013	0.831	0.027	0.029
	SE	0.011	0.001	0.005	0.033	0.013	0.007
Human Differentiated Brown Adipocytes	12h sample 1	0.115	0.032	0.021	0.510	0.022	0.032
	12h sample 2	0.061	0.020	0.016	0.743	0.021	0.011
	12h sample 3	0.017	0.017	0.003	0.580	0.001	0.022
	Ave	0.064	0.023	0.013	0.611	0.015	0.022
	SE	0.028	0.005	0.005	0.069	0.007	0.006
	24h sample 1	0.015	0.005	0.007	0.620	0.006	0.012
	24h sample 2	0.078	0.015	0.008	0.823	0.044	0.014
	24h sample 3	0.059	0.028	0.013	0.697	0.024	0.035
	Ave	0.051	0.016	0.010	0.713	0.025	0.021
	SE	0.019	0.007	0.002	0.059	0.011	0.007
	48h sample 1	0.064	0.026	0.013	0.793	0.041	0.063
	48h sample 2	0.054	0.014	0.008	0.790	0.026	0.027
	48h sample 3	0.029	0.010	0.008	0.767	0.023	0.045
	Ave	0.049	0.016	0.009	0.783	0.030	0.045
	SE	0.010	0.005	0.002	0.008	0.006	0.011

Media sample	Unit:nmol/L									
	**EPA**									
		CYPs				SEH				
Group Info.	Sample ID	17(18)-EpETE	14(15)-EpETE	11(12)-EpETE	8(9)-EpETE	17,18-DiHETE	14,15-DiHETE	11,12-DiHETE	8,9-DiHETE	5,6-DiHETE
Murine Brown Pre-adipocytes	12h sample 1	0.037	#VALUE!	#VALUE!	#VALUE!	2.187	0.075	0.036	0.104	0.009
	12h sample 2	0.068	#VALUE!	#VALUE!	#VALUE!	1.993	0.063	0.036	0.095	0.005
	12h sample 3	0.037	#VALUE!	#VALUE!	#VALUE!	1.703	0.068	0.034	0.090	0.011
	Ave	0.047	#VALUE!	#VALUE!	#VALUE!	1.961	0.069	0.035	0.096	0.009
	SE	0.010	#VALUE!	#VALUE!	#VALUE!	0.140	0.003	0.001	0.004	0.002
	24h sample 1	0.060	#VALUE!	#VALUE!	#VALUE!	2.757	0.091	0.041	0.100	0.007
	24h sample 2	0.046	#VALUE!	#VALUE!	#VALUE!	2.413	0.084	0.030	0.069	0.006
	24h sample 3	0.074	#VALUE!	#VALUE!	#VALUE!	2.117	0.079	0.032	0.081	0.006
	Ave	0.060	#VALUE!	#VALUE!	#VALUE!	2.429	0.084	0.034	0.083	0.007
	SE	0.008	#VALUE!	#VALUE!	#VALUE!	0.185	0.003	0.003	0.009	0.000
	48h sample 1	0.030	#VALUE!	#VALUE!	#VALUE!	3.090	0.138	0.045	0.031	0.006
	48h sample 2	0.048	#VALUE!	#VALUE!	#VALUE!	2.757	0.130	0.043	0.030	0.006
	48h sample 3	0.047	#VALUE!	#VALUE!	#VALUE!	2.980	0.131	0.045	0.044	0.005
	Ave	0.042	#VALUE!	#VALUE!	#VALUE!	2.942	0.133	0.044	0.035	0.005
	SE	0.006	#VALUE!	#VALUE!	#VALUE!	0.098	0.003	0.001	0.005	0.000
Murine Differentiated Brown Adipocytes	12h sample 1	0.090	#VALUE!	#VALUE!	#VALUE!	2.337	0.089	0.044	0.092	0.012
	12h sample 2	0.035	#VALUE!	#VALUE!	#VALUE!	2.940	0.088	0.038	0.111	0.010
	12h sample 3	0.053	#VALUE!	#VALUE!	#VALUE!	2.503	0.091	0.040	0.109	0.014
	Ave	0.059	#VALUE!	#VALUE!	#VALUE!	2.593	0.089	0.041	0.104	0.012
	SE	0.016	#VALUE!	#VALUE!	#VALUE!	0.180	0.001	0.002	0.006	0.001
	24h sample 1	0.036	#VALUE!	#VALUE!	#VALUE!	3.967	0.114	0.047	0.071	0.011
	24h sample 2	0.084	#VALUE!	#VALUE!	#VALUE!	3.290	0.103	0.038	0.089	0.015
	24h sample 3	0.086	#VALUE!	#VALUE!	#VALUE!	3.800	0.090	0.036	0.076	0.005
	Ave	0.069	#VALUE!	#VALUE!	#VALUE!	3.686	0.102	0.040	0.079	0.010
	SE	0.016	#VALUE!	#VALUE!	#VALUE!	0.204	0.007	0.004	0.005	0.003
	48h sample 1	0.194	#VALUE!	#VALUE!	#VALUE!	6.500	0.173	0.070	0.075	0.007
	48h sample 2	0.113	#VALUE!	#VALUE!	#VALUE!	4.700	0.118	0.052	0.062	0.009
	48h sample 3	0.040	#VALUE!	#VALUE!	#VALUE!	4.533	0.123	0.049	0.058	0.009
	Ave	0.116	#VALUE!	#VALUE!	#VALUE!	5.244	0.138	0.057	0.065	0.008
	SE	0.044	#VALUE!	#VALUE!	#VALUE!	0.630	0.018	0.006	0.005	0.001
Human Brown Pre-adipocytes	12h sample 1	0.107	#VALUE!	#VALUE!	#VALUE!	1.210	0.032	0.013	0.065	0.007
	12h sample 2	0.067	#VALUE!	#VALUE!	#VALUE!	1.060	0.026	0.017	0.046	0.011
	12h sample 3	0.083	#VALUE!	#VALUE!	#VALUE!	1.277	0.026	0.021	0.039	0.008
	Ave	0.086	#VALUE!	#VALUE!	#VALUE!	1.182	0.028	0.017	0.050	0.008
	SE	0.012	#VALUE!	#VALUE!	#VALUE!	0.064	0.002	0.002	0.008	0.001
	24h sample 1	0.093	#VALUE!	#VALUE!	#VALUE!	1.247	0.028	0.021	0.039	0.007
	24h sample 2	0.114	#VALUE!	#VALUE!	#VALUE!	0.900	0.027	0.022	0.038	0.012
	24h sample 3	0.040	#VALUE!	#VALUE!	#VALUE!	1.007	0.025	0.024	0.038	0.006
	Ave	0.083	#VALUE!	#VALUE!	#VALUE!	1.051	0.027	0.022	0.038	0.008
	SE	0.022	#VALUE!	#VALUE!	#VALUE!	0.103	0.001	0.001	0.000	0.002
	48h sample 1	0.061	#VALUE!	#VALUE!	#VALUE!	1.233	0.041	0.017	0.013	0.009
	48h sample 2	0.036	#VALUE!	#VALUE!	#VALUE!	1.113	0.035	0.022	0.022	0.007
	48h sample 3	0.020	#VALUE!	#VALUE!	#VALUE!	1.213	0.030	0.030	0.024	0.007
	Ave	0.039	#VALUE!	#VALUE!	#VALUE!	1.187	0.035	0.023	0.020	0.008
	SE	0.012	#VALUE!	#VALUE!	#VALUE!	0.037	0.003	0.004	0.003	0.001
Human Differentiated Brown Adipocytes	12h sample 1	0.031	#VALUE!	#VALUE!	#VALUE!	0.820	0.027	0.011	0.008	0.006
	12h sample 2	0.025	#VALUE!	#VALUE!	#VALUE!	1.063	0.017	0.010	0.022	0.004
	12h sample 3	0.018	#VALUE!	#VALUE!	#VALUE!	0.957	0.020	0.011	0.018	0.004
	Ave	0.025	#VALUE!	#VALUE!	#VALUE!	0.947	0.021	0.011	0.016	0.005
	SE	0.004	#VALUE!	#VALUE!	#VALUE!	0.070	0.003	0.000	0.004	0.000
	24h sample 1	0.025	#VALUE!	#VALUE!	#VALUE!	0.850	0.024	0.006	0.011	0.004
	24h sample 2	0.105	#VALUE!	#VALUE!	#VALUE!	0.957	0.027	0.009	0.013	0.010
	24h sample 3	0.060	#VALUE!	#VALUE!	#VALUE!	0.843	0.034	0.015	0.023	0.007
	Ave	0.063	#VALUE!	#VALUE!	#VALUE!	0.883	0.028	0.010	0.015	0.007
	SE	0.023	#VALUE!	#VALUE!	#VALUE!	0.037	0.003	0.003	0.004	0.002
	48h sample 1	0.043	#VALUE!	#VALUE!	#VALUE!	0.983	0.020	0.011	0.016	0.007
	48h sample 2	0.043	#VALUE!	#VALUE!	#VALUE!	0.933	0.028	0.014	0.014	0.009
	48h sample 3	0.033	#VALUE!	#VALUE!	#VALUE!	0.887	0.020	0.008	0.018	0.009
	Ave	0.040	#VALUE!	#VALUE!	#VALUE!	0.934	0.023	0.011	0.016	0.008
	SE	0.003	#VALUE!	#VALUE!	#VALUE!	0.028	0.003	0.002	0.001	0.001

Media sample

Unit:nmol/L

DHA

		CYPs					SEH					
Group Info.	Sample ID	19(20)-EpDPE	16(17)-EpDPE	13(14)-EpDPE	10(11)-EpDPE	7(8)-EpDPE	19,20-DiHDPE	16,17-DiHDPE	13,14-DiHDPE	10,11-DiHDPE	7,8-DiHDPE	4,5-DiHDPE
Murine Brown Pre-adipocytes	12h sample 1	0.750	0.063	0.057	0.096	2.883	1.637	0.140	0.069	0.393	0.065	0.733
	12h sample 2	0.787	0.082	0.049	0.133	2.757	1.507	0.136	0.065	0.373	0.052	0.687
	12h sample 3	0.847	0.076	0.113	0.164	2.940	1.557	0.152	0.060	0.393	0.030	0.703
	Ave	0.794	0.074	0.073	0.131	2.860	1.567	0.143	0.065	0.387	0.049	0.708
	SE	0.028	0.006	0.020	0.020	0.054	0.038	0.005	0.003	0.007	0.010	0.014
	24h sample 1	0.460	0.061	0.065	0.079	2.087	2.623	0.196	0.088	0.320	0.088	0.593
	24h sample 2	0.420	0.040	0.051	0.075	0.830	2.427	0.199	0.086	0.295	0.048	0.603
	24h sample 3	0.370	0.036	0.045	0.085	1.873	2.533	0.175	0.097	0.330	0.064	0.667
	Ave	0.417	0.046	0.054	0.080	1.597	2.528	0.190	0.090	0.315	0.067	0.621
	SE	0.026	0.008	0.006	0.003	0.388	0.057	0.008	0.004	0.010	0.012	0.023
	48h sample 1	0.357	0.050	0.037	0.113	1.090	6.700	0.329	0.150	0.240	0.095	0.407
	48h sample 2	0.350	0.059	0.029	0.074	2.237	6.367	0.317	0.123	0.233	0.088	0.333
	48h sample 3	0.483	0.086	0.086	0.119	2.597	6.133	0.291	0.115	0.209	0.082	0.443
	Ave	0.397	0.065	0.051	0.102	1.974	6.400	0.312	0.129	0.227	0.089	0.394
	SE	0.043	0.011	0.018	0.014	0.454	0.164	0.011	0.011	0.009	0.004	0.032
Murine Differentiated Brown Adipocytes	12h sample 1	1.053	0.097	0.110	0.193	3.313	2.657	0.197	0.110	0.420	0.093	1.117
	12h sample 2	1.077	0.188	0.138	0.251	4.433	2.590	0.189	0.113	0.473	0.111	1.157
	12h sample 3	0.927	0.104	0.081	0.152	3.237	2.383	0.190	0.108	0.457	0.064	1.073
	Ave	1.019	0.130	0.110	0.199	3.661	2.543	0.192	0.110	0.450	0.089	1.116
	SE	0.047	0.029	0.016	0.029	0.387	0.082	0.003	0.001	0.016	0.014	0.024
	24h sample 1	0.537	0.087	0.076	0.193	4.100	4.200	0.222	0.146	0.367	0.147	1.313
	24h sample 2	0.657	0.086	0.086	0.161	3.007	3.700	0.228	0.130	0.393	0.149	1.257
	24h sample 3	0.710	0.153	0.168	0.251	7.333	3.433	0.207	0.134	0.304	0.143	1.393
	Ave	0.634	0.109	0.110	0.202	4.813	3.778	0.219	0.137	0.355	0.146	1.321
	SE	0.051	0.022	0.029	0.026	1.299	0.225	0.006	0.005	0.026	0.002	0.040
	48h sample 1	1.063	0.165	0.186	0.316	8.033	8.067	0.400	0.292	0.470	0.192	2.953
	48h sample 2	0.477	0.089	0.112	0.163	3.367	6.033	0.291	0.179	0.313	0.105	2.083
	48h sample 3	0.783	0.151	0.117	0.185	4.933	6.000	0.283	0.181	0.324	0.117	2.257
	Ave	0.774	0.135	0.139	0.221	5.444	6.700	0.325	0.217	0.369	0.138	2.431
	SE	0.169	0.023	0.024	0.048	1.371	0.683	0.038	0.037	0.050	0.027	0.266
Human Brown Pre-adipocytes	12h sample 1	0.547	0.089	0.124	0.263	4.767	0.617	0.096	0.047	0.200	0.092	0.763
	12h sample 2	0.610	0.113	0.092	0.209	3.103	0.537	0.090	0.049	0.188	0.130	0.663
	12h sample 3	0.657	0.090	0.094	0.209	3.000	0.560	0.073	0.047	0.231	0.103	0.700
	Ave	0.604	0.097	0.103	0.227	3.623	0.571	0.086	0.048	0.206	0.108	0.709
	SE	0.032	0.008	0.010	0.018	0.572	0.024	0.007	0.001	0.013	0.012	0.029
	24h sample 1	0.251	0.080	0.061	0.151	2.523	0.617	0.090	0.043	0.203	0.125	0.933
	24h sample 2	0.267	0.081	0.089	0.131	3.217	0.557	0.084	0.052	0.167	0.051	0.733
	24h sample 3	0.274	0.074	0.068	0.157	3.433	0.600	0.082	0.054	0.193	0.072	0.610
	Ave	0.264	0.078	0.073	0.146	3.058	0.591	0.085	0.050	0.188	0.083	0.759
	SE	0.007	0.002	0.008	0.008	0.274	0.018	0.002	0.003	0.011	0.022	0.094
	48h sample 1	0.124	0.050	0.067	0.096	1.597	0.787	0.133	0.081	0.164	0.147	0.493
	48h sample 2	0.093	0.024	0.049	0.068	1.233	0.727	0.147	0.072	0.157	0.058	0.573
	48h sample 3	0.103	0.033	0.030	0.082	0.810	0.683	0.135	0.074	0.150	0.060	0.447
	Ave	0.107	0.036	0.049	0.082	1.213	0.732	0.138	0.076	0.157	0.089	0.504
	SE	0.009	0.008	0.011	0.008	0.227	0.030	0.004	0.003	0.004	0.029	0.037
Human Differentiated Brown Adipocytes	12h sample 1	0.104	0.045	0.025	0.085	0.590	0.590	0.096	0.053	0.058	0.069	0.277
	12h sample 2	0.074	0.034	0.028	0.066	1.810	0.557	0.067	0.045	0.050	0.085	0.353
	12h sample 3	0.079	0.017	0.007	0.053	1.543	0.557	0.061	0.037	0.038	0.070	0.337
	Ave	0.086	0.032	0.020	0.068	1.314	0.568	0.075	0.045	0.049	0.075	0.322
	SE	0.009	0.008	0.006	0.009	0.370	0.011	0.011	0.005	0.006	0.005	0.023
	24h sample 1	0.156	0.043	0.051	0.105	1.210	0.733	0.074	0.049	0.086	0.118	0.269
	24h sample 2	0.143	0.049	0.062	0.116	0.750	0.760	0.098	0.070	0.091	0.167	0.370
	24h sample 3	0.202	0.062	0.086	0.107	1.723	0.780	0.103	0.066	0.084	0.168	0.407
	Ave	0.167	0.052	0.066	0.109	1.228	0.758	0.092	0.062	0.087	0.151	0.349
	SE	0.018	0.006	0.010	0.003	0.281	0.014	0.009	0.006	0.002	0.016	0.041
	48h sample 1	0.157	0.039	0.037	0.105	1.107	0.667	0.108	0.053	0.100	0.066	0.527
	48h sample 2	0.211	0.035	0.051	0.105	1.820	0.653	0.092	0.051	0.110	0.117	0.473
	48h sample 3	0.100	0.055	0.042	0.069	1.160	0.637	0.091	0.043	0.099	0.141	0.322
	Ave	0.156	0.043	0.043	0.093	1.362	0.652	0.097	0.049	0.103	0.108	0.441
	SE	0.032	0.006	0.004	0.012	0.229	0.009	0.006	0.003	0.004	0.022	0.061
